# Genome-Destabilizing Effects Associated with Top1 Loss or Accumulation of Top1 Cleavage Complexes in Yeast

**DOI:** 10.1371/journal.pgen.1005098

**Published:** 2015-04-01

**Authors:** Sabrina L. Andersen, Roketa S. Sloan, Thomas D. Petes, Sue Jinks-Robertson

**Affiliations:** Department of Molecular Genetics and Microbiology, Duke University School of Medicine, Durham, North Carolina, United States of America; National Institute of Environmental Health Sciences, UNITED STATES

## Abstract

Topoisomerase 1 (Top1), a Type IB topoisomerase, functions to relieve transcription- and replication-associated torsional stress in DNA. We investigated the effects of Top1 on genome stability in *Saccharomyces cerevisiae* using two different assays. First, a sectoring assay that detects loss of heterozygosity (LOH) on a specific chromosome was used to measure reciprocal crossover (RCO) rates. Features of individual RCO events were then molecularly characterized using chromosome-specific microarrays. In the second assay, cells were sub-cultured for 250 generations and LOH was examined genome-wide using microarrays. Though loss of Top1 did not destabilize single-copy genomic regions, RCO events were more complex than in a wild-type strain. In contrast to the stability of single-copy regions, sub-culturing experiments revealed that *top1* mutants had greatly elevated levels of instability within the tandemly-repeated ribosomal RNA genes (in agreement with previous results). An intermediate in the enzymatic reaction catalyzed by Top1 is the covalent attachment of Top1 to the cleaved DNA. The resulting Top1 cleavage complex (Top1cc) is usually transient but can be stabilized by the drug camptothecin (CPT) or by the *top1-T722A* allele. We found that increased levels of the Top1cc resulted in a five- to ten-fold increase in RCOs and greatly increased instability within the rDNA and *CUP1* tandem arrays. A detailed analysis of the events in strains with elevated levels of Top1cc suggests that recombinogenic DNA lesions are introduced during or after DNA synthesis. These results have important implications for understanding the effects of CPT as a chemotherapeutic agent.

## Author Summary

Topoisomerase I (Top1) nicks one strand of DNA to relieve torsional stress associated with replication, transcription and chromatin remodeling. The enzyme forms a transient, covalent intermediate with the nicked DNA and stabilization of the cleavage complex (Top1cc) leads to genetic instability. We examined the effect of Top1 loss or Top1cc stabilization on genome-wide mitotic stability and on mitotic crossovers that lead to loss of heterozygosity (LOH) in budding yeast. The level of Top1cc was elevated using the chemotherapeutic drug camptothecin or a mutant form of the enzyme. Whereas loss of Top1 only destabilized ribosomal DNA repeats, Top1cc accumulation was additionally associated with elevated LOH and genome-wide instability. In particular, the Top1cc greatly elevated copy number variation at the *CUP1* tandem-repeat locus, consistent with elevated sister chromatid recombination. Molecular examination of LOH events associated with the Top1cc was also consistent with generation of recombination-initiating lesions during or after DNA synthesis. These results demonstrate that the use of topoisomerase inhibitors results in widespread genome instability that may contribute to secondary neoplasms.

## Introduction

DNA is a dynamic molecule with ever-changing states of supercoiling, catenation and single/double strandedness. The separation of DNA strands required for transcription and replication generates overwound (positively supercoiled) DNA in front of the corresponding machineries and can impede their progress if not removed [1]. In addition, underwound (negatively supercoiled) DNA is produced behind RNA polymerases, generating single-stranded DNA that is vulnerable to breakage and promotes formation of RNA:DNA hybrids (R-loops) that inhibit transcription elongation [2]. Finally, chromosomes become catenated via structures such as double Holliday junctions during homologous recombination, and sister chromatids are catenated in the wake of replication. Topoisomerases regulate these topological challenges by breaking the phosphodiester backbone via nucleophilic attack, relieving the topological stress by rotation or strand passage, and re-ligating the DNA by a reverse reaction [3].

Topoisomerases are classified as Type I or Type II, which reflects whether one or two DNA strands, respectively, is initially broken. The budding yeast *Saccharomyces cerevisiae* has three DNA topoisomerases; Top1 and Top3 are Type I enzymes while Top2 is a Type II enzyme. The relevant human homologs are TOP1, TOP2α and TOP2β, and TOP3, respectively. Top3 (as a complex with Sgs1 and Rmi1) has been primarily implicated in homologous recombination, whereas Top1 and Top2 have overlapping functions in regulating topological changes associated with replication and transcription [4]. In the absence of Top1, haploid yeast cells are viable and only elevated recombination within the ribosomal DNA (rDNA) gene cluster has been reported [5, 6]. The rDNA cluster is a 1–2 Mb region on Chromosome XII that represents ~10% of the yeast genome and is comprised of 100–200 tandem copies of a 9.1 kb repeat [7]. In the rDNA of *top1*Δ mutants, mitotic recombination is increased, transcription is disrupted, the rDNA-containing chromosome migrates aberrantly in gels, and extrachromosomal rDNA-containing circles are produced [5, 8, 9].

In addition to maintaining genome integrity, topoisomerases can also be a source of instability. It has been demonstrated, for example, that Top1 is required for the production of a unique class of deletions in short tandem repeats, especially in highly transcribed regions [10, 11]. These deletions are the consequence either of Top1 becoming trapped as a cleavage complex (Top1cc) covalently bound to nicked DNA, or of Top1 incision at a ribonucleoside monophosphate misincorporated into DNA [12]. The genome-destabilizing consequences of a stabilized Top1cc make the enzyme of particular interest as a target of chemotherapeutics. The drug camptothecin (CPT) specifically targets Top1, intercalating into and reversibly trapping stabilized cleavage complexes throughout the genome. When present during replication, a Top1cc is converted into a toxic double-strand break (DSB) that can kill rapidly dividing cells. The CPT analogs topotecan and irinotecan are currently used to treat a variety of cancers including lung, ovarian, cervical, and colon cancers [13]. The genome changes produced in cells that survive chemotherapeutic treatment with CPT analogs are of clinical interest because of their potential role in driving secondary neoplasms.

It is clear that CPT treatment of yeast strains elevates genetic instability, but the destabilizing effects of the drug have only been investigated in limited contexts. For example, CPT treatment of a diploid strain heterozygous at the *ADE1* or *ADE2* locus led to increased loss of the wild-type (WT) allele, and CPT additionally elevated heteroallelic gene conversion at *HIS7* [14]. In a subsequent study, CPT treatment was reported to stimulate sister chromatid exchange between truncated *his3* alleles as well as recombination between similar repeats positioned on nonhomologous chromosomes [15]. Finally, haploid strains with the *top1-103* allele (proposed to be a CPT mimetic) exhibited elevated recombination within the naturally occurring rDNA and *CUP1* tandem repeats as well as between engineered *HIS4* direct repeats [16].

In the current study, we examine global genomic consequences of Top1 loss and of conditions that elevate the level of the Top1cc in diploid strains. An elevated frequency of cleavage complexes was produced by treatment of WT cells with CPT or by expression of a CPT-mimetic *top1* mutant allele (*top1-T722A* [17]). Single-nucleotide polymorphism (SNP)-detecting microarrays were used to map positions of mitotic crossovers on the right arm of chromosome IV, and to monitor loss of heterozygosity (LOH) genome wide [18, 19]. Although loss of Top1 stimulated LOH only within the rDNA tandem repeats, the molecular characteristics of gene conversion events associated with mitotic crossovers in single-copy sequences were altered. In contrast, either Top1-T722A expression or CPT treatment of WT cells substantially increased LOH throughout the genome, including that associated with rDNA. CPT treatment of WT or Top1-T772A expression also greatly stimulated copy-number variation (CNV) within the repetitive *CUP1* locus, suggesting that CNV is a frequent consequence of stabilized Top1 cleavage complexes.

## Results

In diploids that are heterozygous for single-nucleotide polymorphisms (SNPs), mitotic recombination events between homologs can be detected as loss of heterozygosity (LOH). Since the breakpoint of an LOH event reflects the location of the DNA lesion that initiates recombination, mapping of LOH events can yield insights into the mechanism of mitotic recombination [20]. In this study, we used diploid yeast strains constructed by mating derivatives of two sequence-diverged haploids (W303-1A and YJM789) that differ by approximately 55,000 SNPs [21]. As will be described further below, LOH events involving ~15,000 SNPs distributed throughout the genome can be monitored using SNP microarrays [18]. In some experiments, we mapped mitotic crossovers selected to occur on the right arm of chromosome IV, a region of ~1 Mb. In a second type of experiment, LOH events throughout the genome were mapped.

### Frequency of crossovers on the right arm of chromosome IV in a *top1/top1* diploid

**Fig 1:**
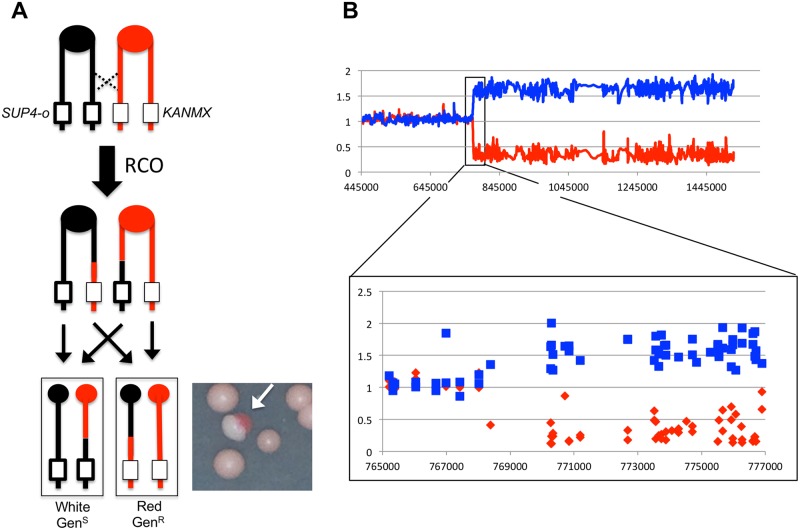
Detecting and characterizing RCOs on chromosome IV. **(A)** JSC25 chromatids shown in red are derived from W303-1A and contain a *KANMX* cassette on the right arm of chromosome IV. Chromatids in black are derived from YJM789 and contain a *SUP4-o* marker on the right arm of chromosome IV. In addition the diploid strain is homozygous for the *ade2-1* mutation. An RCO that leads to LOH generates a red/white sectored colony (white arrow) when the daughter cells segregate. **(B)** Microarray analysis. The W303-1A-related SNP hybridization signal is shown as a red line and that of YJM789 is the blue line. A hybridization value of ~1 indicates a single copy of a given SNP is present (heterozygous SNP), a value between 1.5 and 2 indicates two copies of the SNP, and a value of 0–0.5 indicates no copy of the SNP is present. Shown is the white side of a sector with a transition from heterozygous to YJM789-homozygous SNPs.

The *top1*Δ*/top1*Δ diploid strain (SLA46.D4) used to map LOH events had, in addition to heterozygosity for 55,000 SNPs, markers located near the right end of chromosome IV to allow identification of crossovers. As with JSC25, the isogenic wild-type (WT) diploid used in previous studies [19], SLA46.D4 also had an insertion of the ochre-suppressing *SUP4-o* gene near the right telomere (SGD coordinate 1510386) of chromosome IV on the YJM789-derived homolog and an insertion of a *KANMX* gene, which confers geneticin resistance, inserted at exactly the same position on the W303-1A-derived homolog. Finally, the strain was homozygous for the *ade2-1* ochre allele. Zero, one, or two copies of *SUP4-o* in a diploid *ade2-1/ade2-1* background result in red, pink, or white colonies, respectively. JSC25 and SLA46.D4 thus form pink colonies, but can be screened for red/white sectored colonies that result from reciprocal crossover (RCO) events between *CEN4* and the inserted *SUP4-o*/*KANMX* markers (Fig. 1A). The red portion of a sectored colony contains no copy of *SUP4-o* and is geneticin-resistant, while the white portion contains two *SUP4-o* copies and is geneticin-sensitive. As described below, we first determined the frequency of red/white colonies in SLA46.D4 as a measure of the frequency of RCOs in *top1*Δ strains and then mapped the positions of the crossovers. Because the formation of a red/white sectored colony requires that the recombination event occur at the time of plating, the frequency of sectored colonies is equivalent to a rate measurement. The production of a sectored colony also requires that each daughter cell contain one recombinant chromosome and one non-recombinant chromosome (Fig. 1A). Of 1.3x10^6^ SLA46.D4 colonies that were screened, 35 red/white sectored colonies were identified, giving a sectoring rate of 2.7x10^-5^/division. This rate is not significantly different from that of the WT strain JSC25 (3.1x10^-5^ [19]; p = 0.45), indicating that loss of Top1 does not significantly affect the rate of spontaneous crossovers between homologs.

### Mapping crossovers and associated gene conversion events selected on chromosome IV

The positions of recombination events in W303-1A/YJM789 diploids were mapped using SNP-specific microarrays, the details of which have been described previously [19]. In brief, genomic DNA was isolated from colonies derived from both the red and white sectors. DNA from the experimental samples was labeled using nucleotides containing a fluorescent dye (usually Cy5-dUTP) and mixed with DNA from a control heterozygous strain that had been labeled with a different fluorescent tag (usually Cy3-dUTP). The experimental and control samples were then mixed and competitively hybridized to oligonucleotide-containing microarrays. For the chromosome IV experiments, arrays examined ~2300 SNPs located between *CEN4* and the *SUP4-o/KANMX* markers. Each SNP was represented by four 25-base oligonucleotides; two corresponded to the Watson and Crick strands of the W303-1A allele and two to the Watson and Crick strands of the YJM789 allele. Following hybridization to the SNP microarray, the ratio of Cy3 to Cy5 hybridization to each individual oligonucleotide was determined. After appropriate normalization steps (described in [19]), a Cy3:Cy5 hybridization ratio of 1 at a specific SNP indicated that the experimental strain was heterozygous at that SNP. If, for example, the experimental sample had an LOH event that resulted in homozygosity for the YJM789-derived SNP, hybridization to YJM789-specific oligonucleotides was 1.5- to 2-fold greater than that of the heterozygous control. Concurrently, the same experimental sample had a decreased relative level of hybridization to the W303-1A-specific oligonucleotides, with a ratio of 0.1–0.5 relative to the heterozygous control. These relative hybridization levels show some variation because the specificity of a particular oligonucleotide for one allele or the other is a complex function of the melting temperature of perfectly-matched versus imperfectly-matched duplexes. However, the transition between heterozygous and homozygous SNPs is usually unambiguous, as illustrated by the example shown in Fig. 1B.

**Fig 2:**
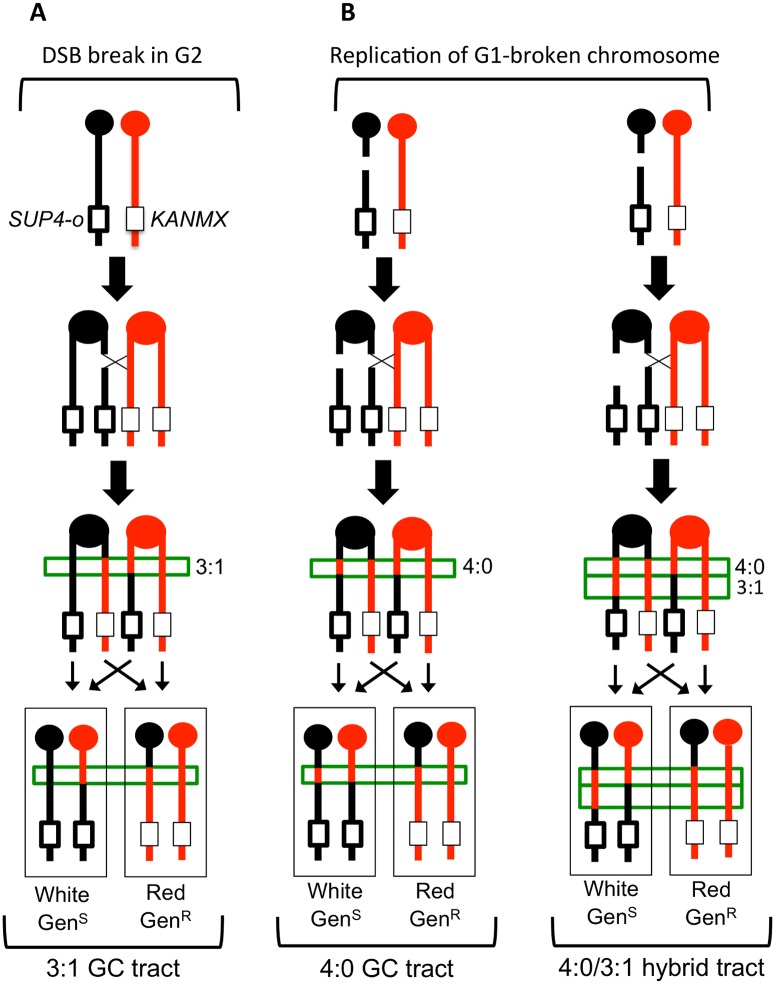
Gene conversion pattern associated with G1- or G2- initiated DSBs. **(A)** A DSB is generated in one sister chromatid after DNA synthesis and repair of the sister chromatid from the homolog results in a 3:1 conversion tract, which is within the green box. **(B)** A DSB occurs in G1 and the chromosome is replicated to form two broken sister chromatids. Repair of the two broken chromatids from the homolog results in a 4:0 conversion tract. On the left, the two conversion tracts are the same length. On the right, the lengths of the gene conversion tracts differ, giving rise to a hybrid 3:1/4:0 event. W303-1A and YJM789 chromatids are red and black, respectively.

A comparison of LOH transition points in the red and white sectors yields additional information about the nature of the recombination event [18–20, 22, 23]. In previous studies in the WT strain JSC25, the LOH transitions in 13% of the sectored colonies were identical in the red and white sectors (“simple” CO). In the majority of the samples, however, the transition points were different. An example is illustrated in Fig. 2A, where the LOH transition in the red sector occurs centromere-proximal to the transition in the white sector. Considering all four chromosomes in the two sectors, there is a region (boxed in green) in which three of the chromosomes have red (W303-1A) SNPs and one has black (YJM789) SNPs. This 3:1 pattern signals a mitotic gene conversion (GC) associated with the selected crossover event [18, 19]. A GC reflects repair of the broken chromosome via a non-reciprocal transfer of DNA sequence from the intact chromosome, and occurs near the site of the initiating lesion [24]. The transfer of information often involves heteroduplex formation, followed by repair of the resulting mismatches within the heteroduplex [25]. GC events in yeast are associated with crossovers about 40% of the time [26], and ~90% of crossovers are associated with a detectable GC event [19]. Mitotic conversion tracts are variable in length, ranging from less than 100 bp to more than 50 kb [26], with a median length of 10.6 kb for spontaneous conversion events [19].

The pattern of LOH in Fig. 2A is consistent with the repair of a single chromatid broken in S or G2 of the cell cycle. We also observed events that were consistent with the repair of two sister chromatids broken at approximately the same position (Fig. 2B). In such events, there is a region adjacent to the crossover in which all four chromosomes have SNPs derived from one homolog (a 4:0 GC tract; Fig. 2B, left). One simple interpretation of these events is formation of a DSB in G1, with subsequent replication of the broken chromosome to yield broken sister chromatids [19, 20, 22]. In addition to 4:0 GC tracts, 4:0/3:1 hybrid conversion tracts were observed. These tracts also likely initiate with a G1-associated DSB, with the two broken chromatids repaired to generate different sized conversion tracts (right side of Fig. 2B). In summary, an analysis of a sectored colony by microarrays allows: (1) mapping the position of the crossover, (2) measurement of GC tract lengths associated with the crossover, (3) determination of which homolog had the initiating DNA lesion, and (4) inference about the timing of the initiating DNA lesion.

**Fig 3:**
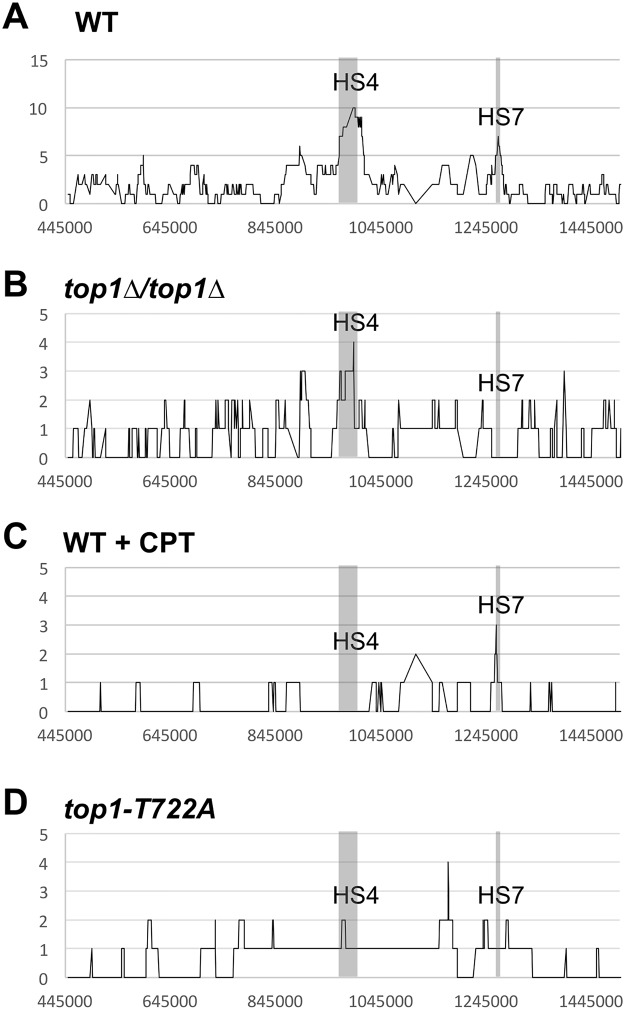
Distributions of LOH breakpoints. Plotted is the number of times each SNP was included in a crossover-associated conversion tract (Y-axis) and the SGD coordinates for chromosome IV (X-axis) in **(A)** WT, **(B)** *top1Δ*/*top1Δ*, **(C)** WT + CPT, and **(D)** *top1-T722A* sectors. Hotspots 4 (HS4: Chr IV SGD coordinates 970-1000kb) and 7 (HS7: Chr IV SGD coordinates 1260-1270kb) were previously noted in a WT background [18] are indicated by the shaded region.

We mapped LOH events in both the red and white portions of 88 sectored colonies identified in the *top1*Δ/*top1*Δ background (SLA46.D4) by SNP microarrays and compared these with 139 sectors we previously characterized in the WT strain JSC25 [19]. Although the rate of spontaneous crossovers on chromosome IV was not affected by Top1 loss, the distribution and types of events were different. The distributions of events in the WT and *top1*Δ/*top1*Δ strains are shown in Fig. 3A and B, respectively. In both strains, events were widely distributed in the 1 Mb interval between *CEN4* and the *SUP4-o/KANMX* markers. In our previous analysis of the wild-type strain, we identified seven peaks of recombination activity, termed HS1-HS7 [19]; two of these peaks are labeled in Fig. 3A. The prominent HS4 hotspot, which has closely-spaced, inverted Ty retrotransposons and was previously identified in the WT strain [19], was also evident in the *top1*Δ strain. By contrast, the HS7 hotspot seen in the WT strain was not present in the *top1*Δ strain. This HS7 region was included in a conversion tract in seven of the 139 WT sectors analyzed, but was not represented among conversion tracts in the 88 sectors isolated in the absence of Top1 (p = 0.04 by Fisher exact test). By the same type of analysis, there were no significant differences between the WT and *top1*Δ strains for HS1-HS3, HS5, or HS6. The reason that HS7 is an apparent Top1-dependent hotspot is not apparent from the genes and sequence motifs in the relevant region (SGD coordinates 1265–1275 kb).

In previous studies, we found non-random associations between LOH breakpoints and certain elements of chromosome structure [18, 22]. Previous analysis of breakpoints in the WT strain detected enrichment for Ty elements, long terminal repeats (δ elements) that flank Ty elements, and tRNA genes [19], whereas a reduction in the level of DNA polymerase α induced instability at regions associated with replication pausing, such as replication-termination (TER) regions [23]. We performed a similar analysis of LOH breakpoints in the *top1*Δ strain (details in Supplementary Information). There was no significant enrichment or exclusion of palindromes, tandem repeats, triplet repeats, G4 DNA motifs, tRNA genes, intron-containing genes, autonomously replicating sequence (ARS) elements, weakly- or highly-transcribed genes, Ty elements, δ elements not associated with Ty, γ-H2AX foci, Rrm3 pause sites, high GC content, TERs, or ARS elements flanked by opposing transcripts. The failure to detect associations seen in the WT background could either reflect the Top1-dependence of the initiating DSBs in these regions or simply reflect the smaller number of sectored colonies examined.

As described above, the type of gene conversion tract associated with an RCO event provides information about when the initiating DNA damage occurred during the cell cycle. Of the 88 sectored *top1*Δ colonies examined, 10 had no associated conversion event, 23 had a 3:1 conversion tract, two had a 4:0 conversion tract, and 12 had a hybrid 3:1/4:0 conversion tract. The remaining 41 conversion events were complex, with multiple transitions between heterozygous and homozygous markers or transitions between homozygous markers derived from different homologs. The coordinates for these transitions are in S1 Table and all conversion events are schematically depicted in S2 Table. Excluding the complex tracts, the *top1*Δ strain had 14 G1-like conversions and 23 G2-like conversions, compared to 53 G1-like conversions and 29 G2-like conversions for the WT strain [19]. The proportions of G1- versus G2-like events are significantly different (p<0.01 by Fisher exact test).

Of the 78 conversions in the *top1*Δ strain, 41 (53%) were complex; in the WT strain, only 39 of 121 conversions (32%) were complex [19]. These proportions are significantly different (p = 0.005, Fisher exact test). By a variety of criteria (see Supplementary Materials and Methods), complex events can be classified as either G1 or G2. The most common criterion we used is that complex events with at least one 4:0 region were considered G1 events. Among the complex events there were 35 G1-like events and six G2-like events in the *top1*Δ strain, compared to 37 G1-like and 2 G2-like events in WT [19]. If we sum the G1 and G2 events for simple and complex conversions, the *top1*Δ strain had 49 G1 and 29 G2 events, and the WT strain had 90 G1 and 31 G2 events. By Fisher exact test, these numbers are not significantly different (p = 0.11).

Based on the coordinates of homozygous to heterozygous SNP transitions in sectored colonies (S1 Table), we calculated the lengths of gene conversion tracts in the *top1*Δ diploid. Each tract length was calculated by averaging the maximum length (the difference between the coordinates of the SNPs that most closely flanked, but were not within, the tract) and the minimum length (the coordinates of the SNPs that were within the tract at the termini of the conversion event). For more complex tracts with multiple transitions, the tract lengths were calculated using only the first and last transitions. Finally, because it is likely that most crossovers are associated with conversion events, we also calculated conversion tract lengths for those crossovers that did not have a detectable conversion by averaging the minimal possible conversion tract (1 bp) and the maximal tract (the distance between the heterozygous and homozygous SNPs closest to the breakpoint).

The median tract length for all conversion tracts in the *top1*Δ strain was 13.6 kb, compared to a value of 10.6 kb for WT [19]. By the Mann-Whitney test, the distribution of tract lengths was significantly different (p = 0.002). By the same statistical test, the medians of the G1-associated conversion tracts (14.8 kb for WT and 17.7 kb for *top1*Δ) were not significantly different (p = 0.1), but the medians for the G2-associated conversion tracts (4.7 kb and 9.6 kb for WT and *top1*Δ, respectively) were different (p<0.001).

### Unselected LOH events in *top1*Δ diploid strains

In the experiments described above, we used microarrays containing oligonucleotides specific to chromosome IV. Four red/white SLA46.D4 (*top1*Δ*/top1*Δ) sectored colonies were also examined using whole-genome microarrays that detect unselected LOH and gene copy-number variation throughout the entire genome at ~1 kb resolution [18]. Of the four red/white sectors analyzed, three had terminal LOH events on chromosome XII with a breakpoint in the rDNA. One sectored colony was consistent with a break-induced replication (BIR) event, with half of the sector remaining heterozygous distal to the rDNA, and the other half homozygous for the W303-1A-derived SNPs located centromere-distal to the rDNA locus. The other two sectored colonies had identical terminal LOH events in both the red and white sectors; all sectors were homozygous distal to the rDNA locus for the YJM789-derived SNPs. These events could represent crossovers within the rDNA or BIR events that occurred before plating. It should be noted that our microarrays do not have oligonucleotides representing SNPs within the rDNA and, therefore, we cannot detect gene conversion events within the cluster. Other than the terminal LOH events initiating in the rDNA, there were no unselected LOH events detected on other chromosomes.

Since the frequency of unselected LOH events in the sectored colonies was very low, we also examined ten independent SLA46.D4 colonies that had been sub-cultured (colony to colony) ten times (~250 generations) to allow LOH events to accumulate prior to analysis via whole-genome microarrays. Among the ten SLA46.D4 sub-cultured lines, nine had a terminal LOH on chromosome XII with a breakpoint in the rDNA. In one line, there was an additional interstitial LOH event (gene conversion) on chromosome XV. In summary, the whole-genome analyses support our previous conclusion (based on sectoring rates) that *top1*Δ mutants do not have substantially elevated levels of mitotic recombination, except within the rDNA.

### Mitotic crossovers are elevated in strains that accumulate Top1 cleavage complexes

**Fig 4:**
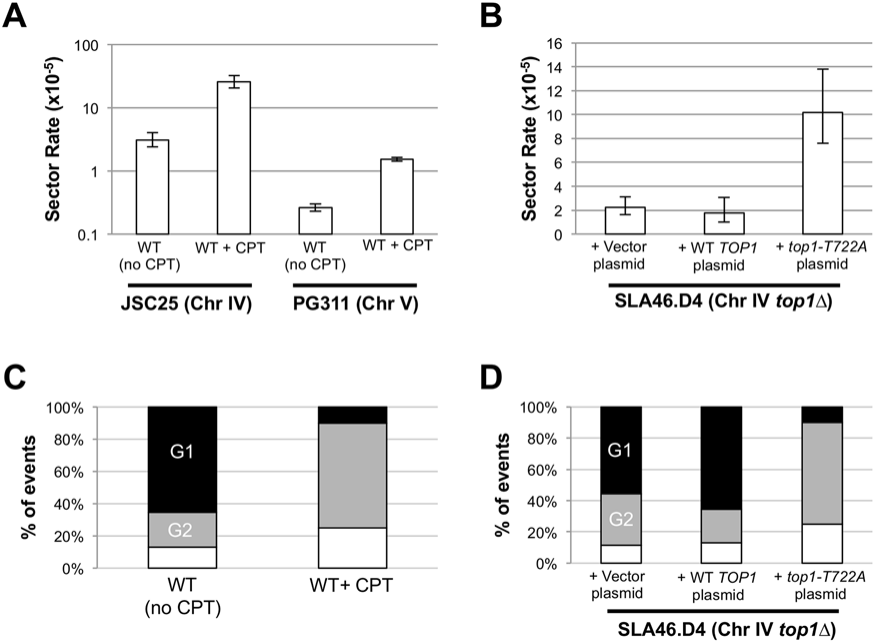
CPT treatment and Top1-T722A expression increase RCOs during or after DNA synthesis. Rates of red/white sectoring associated with **(A)** CPT treatment or **(B)** Top1-T722A expression are shown. The cell cycle phase when an RCO-initiating event occurred was inferred by the associated gene conversion pattern: **(C)** CPT treatment or **(D)** Top1-T722A expression stimulates initiation primarily in G2. Black, G1-associated; gray, G2-associated; white, simple CO (no GC).

We examined the genome-destabilizing effects of Top1 cleavage complexes (Top1cc) generated by two different approaches: (1) use of camptothecin (CPT), a drug that stabilizes the Top1cc and (2) use of a specific *top1* mutant (*top1-T722A*) that accumulates Top1 cleavage complexes [17]. It should be noted that cytotoxic effects of CPT are completely suppressed by deletion of *TOP1*, indicating that Top1 is the only *in vivo* target of the drug [27]. In CPT experiments, we incubated the WT diploid strain JSC25 for six hours in either rich growth medium containing dimethyl sulfoxide (DMSO) and 500 μM CPT, or medium containing DMSO. As described previously, JSC25 has *SUP4-o/KANMX* markers near the end of the right arm of chromosome IV, allowing the detection of RCOs as red/white sectored colonies (Figs. 1 and 2). As shown in Fig. 4A, CPT treatment elevated the frequency of RCOs about eight-fold. We found 79 sectors among 3.1 x 10^5^ total colonies derived from CPT-treated cells and four sectored colonies in 1.3 x 10^5^ total colonies derived from the DMSO-treated control cells; this difference is significant (p<0.001 by a chi-square test). The rate of sectors in the DMSO-treated control cells is about the same as in untreated JSC25 cells [19], although we note that the former rate is based on a small number of sectored colonies.

To better quantitate the effect of CPT on crossovers, we used a related diploid strain (PG311) in which crossovers on the left arm of chromosome V can be more easily detected [20]. In this strain, one homolog has the *can1-100* ochre allele located ~33 kb from the left end of chromosome V; on the other homolog, the *CAN1* gene was replaced with the ochre-suppressing *SUP4-o* allele. The diploid is also homozygous for the *ade2-1* ochre mutation. Crossovers between *CEN5* and the *can1-100/SUP4-o* markers result in canavanine-resistant red/white colonies ([20, 28]; S1 Fig). Because only cavananine-resistant colonies of PG311 and its derivatives need to be screened for red/white sectors (as opposed to all JSC25 colonies), we were able to obtain a more accurate measurement of the rate of RCOs in the control and experimental strains. We found that treatment of PG311 with CPT elevated the rate of crossovers ~ six fold relative to the untreated control (Fig. 4A). We observed 190 Can^R^ red/white sectored colonies (total of 7.3 x 10^7^ cells plated) in the control strain compared to 761 sectored colonies (total of 5 x 10^7^ cells plated) in the CPT-treated sample. This difference is significant (p = 0.001) by the chi-square test. As expected by the relative of size of the intervals monitored on chromosomes IV and V, there were ~10-fold more RCOs on chromosome IV.

To examine the effect of Top1-T722A expression on RCOs on the right arm of chromosome IV, we transformed the *top1*Δ/*top1*Δ strain (SLA46.D4) with pWJ1490, which contains the coding sequence of the *top1-T722A* allele fused to the copper-inducible *CUP1* promoter (*pCUP1*; [29]). As controls, SLA46.D4 was transformed with empty vector (pRS416) or with a *pCUP1-TOP1* construct (pWJ1491). Because addition of copper to the plasmid-selective growth medium was not required for induction of the *pCUP1-top1-T722A* allele [29], experiments were performed without addition of exogenous copper. Expression of the *top1-T722A* allele elevated the red/white sector rate approximately five-fold (p<0.0001) relative to cells containing either empty vector or the *pCUP1*-*TOP1* allele (Fig. 4B).

### Characterization of recombination events associated with stabilized Top1 cleavage complexes

The positions and types of crossover-associated gene conversion events on right arm of chromosome IV were mapped in 20 CPT-induced sectors (S3 and S4 Tables) and in 20 sectors isolated during expression of the mutant Top1-T722A protein (S5 and S6 Tables), and these profiles were compared to those previously reported for WT [19]. The prominent HS7 found in WT, but absent in the *top1*Δ/*top1*Δ background, was not affected in CPT-treated JSC25 (Fig. 3C; p = 0.11) or in the strain expressing the Top1-T722A protein (Fig. 3D; p = 0.6). Likewise, there was no significant effect of CPT treatment or Top1-T722A expression on HS4 (p = 0.36 and p = 1 by Fisher exact test).

As shown in Fig. 2, RCOs associated with simple 3:1 conversion tracts are consistent with a DSB affecting only one sister chromatid (G2 event), while those with simple 4:0 or hybrid 4:0/3:1 tracts presumably reflect two broken sister chromatids (G1 event). In the WT and *top1*Δ strains, most of the conversions (74% and 63%, respectively) were G1-like events. Following CPT treatment of JSC25, however, there were only two G1-like crossover-associated conversion events and 13 were G2-like (S3 and S4 Tables). An additional five events were not associated with conversion tracts and could not be classified as G1- or G2-like. In the strain expressing the *top1-T722A* allele, there similarly were two G1-like events, 13 G2-like events and 5 simple crossovers with no associated gene conversion tract (S5 and S6 Tables). For both the CPT-treated cells and the cells expressing the Top1-T722A protein, the differences in the number of G1- and G2 events was significant when compared to WT (p<0.001 for both comparisons). A summary of the cell-cycle distributions of recombinogenic lesions is shown in Fig. 4C-D. These observations suggest that stabilized Top1 cleavage complexes result in single-chromatid breaks and, as discussed further below, it is likely that these breaks are associated with DNA replication.

The frequencies of conversion tracts that were complex in CPT-treated and *top1-T722A* strains were 30% and 7%, respectively, and these tracts were included in the G1 versus G2 analysis described above. Neither complex frequency was significantly different from the frequency observed in the untreated WT strain. The median lengths of conversion tracts in the CPT-treated strain were 24.8 kb (G1), 4.7 kb (G2) and 7.2 kb (all conversions); by the Mann-Whitney test, none were different from the corresponding tract lengths in WT (14.8 kb, 4.7 kb and 10.6 kb, respectively; [19]). In the *top1-T722A* strain, the median conversion tract lengths were 18.8 kb (G1), 9.3 kb (G2) and 9.3 kb (all). The lengths of the G2-associated and total conversions were significantly different from WT at p values of 0.04 and 0.05, respectively. The G2-associated events included two very large conversion tracts of ~100 kb and 400 kb, which are much larger than most tracts in previous studies [19, 20]. These two events could represent false sectors and if removed from the dataset, G2-like and total conversion tract-length differences in WT and *top1-T722A* strains were no longer significant.

### Genome-wide LOH and copy-number changes associated with Top1cc

In addition to examining recombination events on chromosome IV, we analyzed unselected events induced by CPT+DMSO throughout the genome, as well as events in a WT control strain (JSC25) treated with DMSO only. Each isolate was sub-cultured ten times (~250 generations) and extracted DNA was examined using whole-genome microarrays; results are summarized in S7 and S8 Tables. In two DMSO-treated JSC25 isolates, we found two alterations: one interstitial and one terminal LOH event. Interstitial events presumably correspond to a conversion event, while terminal LOH events could reflect either a crossover or a BIR event. In a larger sample of 13 WT isogenic isolates sub-cultured twice without CPT or DMSO, only one LOH event was observed [18]. Thus, in WT strains there were ~0.5 LOH events per isolate after the equivalent of ten sub-cultures.

Four isolates of JSC25 were sub-cultured ten times on plates containing DMSO plus 500 μM CPT. One CPT-treated isolate had no detectable LOH events; one had a terminal LOH event on chromosome XII with a breakpoint in the rDNA locus; one had an interstitial and a terminal LOH event; and one had two interstitial and three terminal LOH events. The average number of LOH events per isolate in the presence of CPT was ~2 and thus about four times higher than in the untreated isolates.

Using the same approach, we examined the effect of Top1-T722A expression on genome stability. In four independent isolates of SLA46.D4 that were sub-cultured ten times, we found a total of five interstitial LOH events, one terminal LOH event, and two deletions, resulting in an average of two LOH events per isolate. In addition, as described below, all four sub-cultured lines had copy-number variation at the *CUP1* locus.

### Copy-number variation (CNV) at the *CUP1* locus is elevated by CPT treatment or Top1-T722A expression

**Fig 5:**
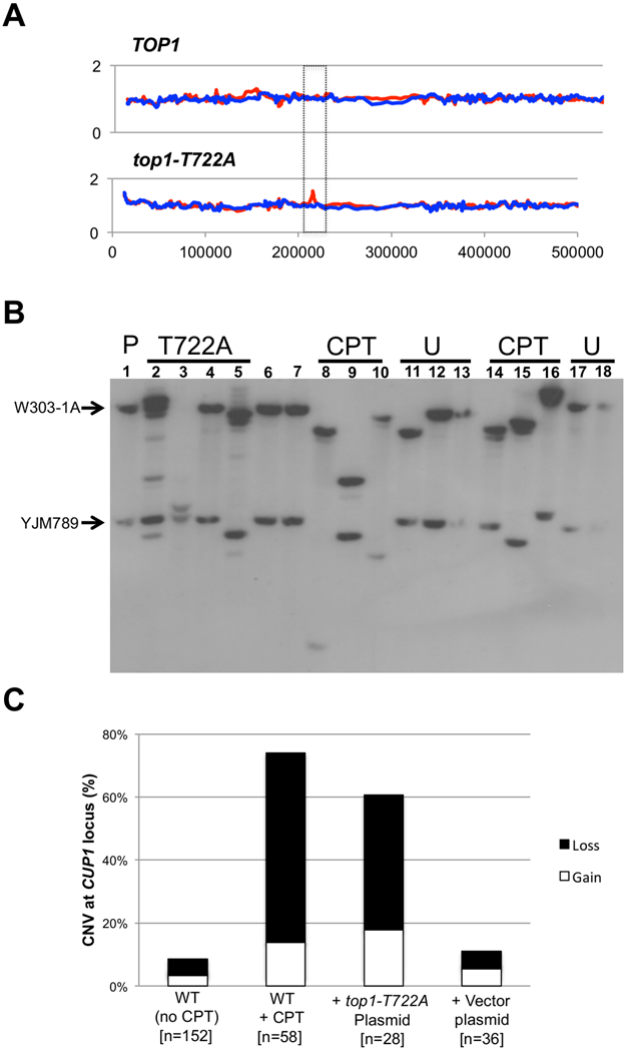
*CUP1* instability associated with CPT treatment or Top1-T722A expression. Panel **(A)** illustrates an increase in the number of *CUP1* repeats on the W303-1A allele (red peak in boxed region) as detected by microarrays. In **(B)** a representative *CUP1* Southern blot is shown. DNA isolated from cells sub-cultured 10 times (~250 generations) was digested with *Eco*RI and the size of the array on each homolog was examined by Southern blot using a *CUP1*-specific probe. The arrows indicate the sizes of the *CUP1* arrays in the parent. The most robust band was used as the measure for gain or loss. **(C)** CPT treatment or Top1-T722A expression lead to a significant increase in copy number variation (CNV) after 10 sub-cultures. Also shown is the percentage of alleles that lose (black bars) compared to alleles that gained *CUP1* repeats (white bars).

In each of the four sub-cultured *top1-T722A* isolates, we observed changes in the number of *CUP1* genes by microarray analysis; three increases (an example is shown in Fig. 5A) and one reduction in the W303-1A-specific SNPs were detected. The *CUP1* locus is a naturally occurring tandem array of the metallothionein-encoding *CUP1* gene on chromosome VIII. Different yeast strains have variations both in the total number of *CUP1* repeats and in the size of the repeat unit [30–33]. The W303-1A-derived homolog has 14 copies of a 2-kb repeat whereas the YJM789-derived homolog has seven copies of a 1.2 kb repeat [30]. On the whole-genome microarray, the *CUP1* locus is represented by oligonucleotides that are specific to the W303-1A repeat [18]. Thus, we can detect changes in the number of repeats only on the W303-1A homolog. In addition, although microarrays can easily detect changes in copy number that involve five or more repeats, smaller changes would be difficult to detect. For these reasons, we examined alterations in the number of *CUP1* repeats by Southern analysis.

The restriction enzyme *Eco*RI has no recognition sites within either the 1.2 or 2.0 kb repeats, but cuts within sequences that closely flank the repeats [30]. In the parental diploid, treatment of genomic DNA with *Eco*RI, followed by Southern analysis with a *CUP1*-specific probe, produced two fragments of about 30 kb (the W303-1A array) and 12 kb (the YJM789 array). Southern analysis confirmed the CNV detected by the microarrays in the four *top1-T722A* sub-cultured clones, and additionally revealed CNV in the three of the four sub-cultured WT clones treated with CPT.

To more accurately quantitate *CUP1* instability resulting from Top1 cleavage complexes, we sub-cultured additional isolates of the WT strain with and without CPT treatment, the *top1-T772A* strain, and the *top1*Δ/*top1*Δ diploid strain. As shown in Fig. 5B, both deletions and additions of *CUP1* repeats were observed. In some isolates, only one array was altered (Fig. 5B, lane 3) whereas, in others (Fig. 5B, lane 9), both arrays changed in size. In addition, some genomic samples had more than two *CUP1*-hybridizing DNA fragments (Fig. 5B, lane 2). These additional fragments likely represent deletions and additions that occurred in the final sub-culturing. This was confirmed by examining the *CUP1* locus after the first subculture, at which point faint fragments were frequently evident in isolates expressing Top1-T722A or treated with CPT (S9 Table). Our conclusions about the nature of copy number changes after ten sub-cultures were based on analysis only of the two fragments that hybridized most strongly to the *CUP1* probe. When both arrays were altered in size, we assume that the larger of the new fragments was from the W303-1A-derived homolog and the smaller from the YJM789-derived homolog.

Changes in the number of *CUP1* repeats are summarized in Fig. 5C, where we show the percentage of chromosome VIII homologs with changes in *CUP1* repeat number after ten rounds of sub-culturing; the complete data set is presented in S9 Table. Control data were derived from two nearly-isogenic *TOP1*/*TOP1* strains (JSC25 and PG311) grown either in rich medium or in rich medium containing DMSO. Thirteen of the 152 homologs analyzed (9%) from the control strains had alterations in the number of *CUP1* repeats. In strain JSC25 treated with CPT, 43 of the 58 homologs (74%) had changes in the number of *CUP1* repeats. The *top1-T722A-*expressing strain had similarly high levels of *CUP1* instability, with 17 of 28 (61%) homologs exhibiting CNV. The level of instability for the *top1*Δ strain was no different than in the WT control (p = 0.75).

For the strains examined in our study, the number of deletions generally exceeded the number of additions (Fig. 5C). The WT control strains had eight deletions and five additions (p = 1); the sums of all alterations for the CPT-treated and the Top1-T722A-expressing strains were 47 deletions and 13 additions (p = 0.002). As discussed further below, the deletion bias is relevant to the likely mechanism of repeat instability at *CUP1*. Our demonstration that CPT treatment and expression of Top1-T722A leads to elevated rates of deletions and duplications in *CUP1* is consistent with previous results showing that the *top1-103* mutation increases the rate of loss of *URA3* integrated into the *CUP1* array [16].

### Crossovers at the rDNA locus increase with CPT treatment and Top1-T722A expression

In the *top1*Δ/*top1*Δ strain SLA46.D4, three of four red/white sectored colonies and nine of ten sub-cultured clones had LOH events with breakpoints at the rDNA locus (see above). To better quantitate the effect of Top1 on rDNA stability and to examine possible rDNA instability associated with the Top1cc, we monitored a restriction site polymorphism (a *Spe*I site present in YJM789 but absent in W303-1A) located ~21 kb centromere-distal to the rDNA locus. Using primers that flank the polymorphism, we PCR-amplified genomic DNA and then treated the resulting fragment with *Spe*I. Heterozygous strains have three DNA fragments of ~750, 500 and 250 bp. Strains homozygous for the YJM789 allele produce only the 500 and 250 bp fragments, while those homozygous for the W303-1A allele produce only the 750 bp fragment.

**Fig 6:**
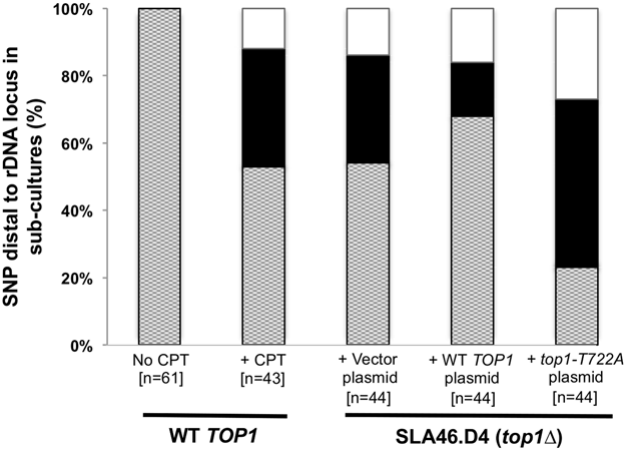
CPT treatment and Top1-T722A expression result in increased RCOs at the rDNA locus. An rDNA-distal PCR product from sub-culture 10 clones was digested with *Spe*I and analyzed by gel electrophoresis. Dotted, heterozygous; black, homozygous for W303-1A; white, homozygous for YJM789.

We sub-cultured strains of various genotypes ten times, and then examined them for LOH of the *Spe*I polymorphism (Fig. 6 and S10 Table). Among the 61 isolates of WT strain JSC25 examined, no LOH events were detected. In contrast, 20 of 44 *top1*Δ/*top1*Δ diploids had an LOH event. These results confirm our microarray analyses as well as the conclusion of previous studies [5]. CPT treatment of JSC25 greatly stimulated inter-homolog mitotic crossovers on chr. XII, with 20 of 43 clones exhibiting LOH (p<0.0001 relative to control). In addition, in strains expressing the *top1-T722A* mutant allele, 34 of 44 sub-cultures had LOH for the *Spe*I polymorphism. Unexpectedly, the *top1*Δ strain containing the plasmid-borne *TOP1* gene (SL146.D4/pWJ1491) also exhibited an elevated level of LOH relative to WT JSC25, with 14 of 44 isolates exhibiting LOH. This frequency of LOH is significantly lower than that observed in strains expressing Top1-T722A (p<0.0001), and similar to that observed in the *top1*Δ strain with the vector (p = 0.27). Although this suggests that the plasmid-borne *TOP1* gene likely fails to fully complement the chromosomal deletion, it also is possible that overexpression of Top1 from the *CUP1* promoter might drive instability.

## Discussion

The main conclusions of this study are: (1) loss of Top1 does not significantly elevate the frequency of inter-homolog crossovers except at the rDNA locus, (2) elevated levels of Top1 cleavage complexes, resulting from CPT treatment or expression of the *top1-T722* allele, stimulate both inter-homolog recombination and inter-sister chromatid recombination, and (3) the recombinogenic DNA lesions caused by the Top1 cleavage complexes likely induce recombination in S or G2. Each of these conclusions will be discussed further below.

The Top1 topoisomerase has a variety of roles in the yeast, including preventing the accumulation of positive supercoils in front of the transcription and replication machineries, and preventing accumulation of negative supercoils behind transcribing RNA polymerase. It is thus difficult to predict the effects of removing Top1 on recombination. Loss of Top1, for example, might induce recombination because accumulation of supercoils leads to stalled replication forks or because R-loops give rise to recombinogenic DNA lesions. Alternatively, some spontaneous mitotic recombination events might be a consequence of Top1-induced nicks that, if unrepaired, lead to recombinogenic double-stranded breaks (DSBs). The latter mechanism is responsible for the hyper-recombination (hyper-rec) phenotypes of strains defective in RNase H2, which initiates removal ribonucleotides embedded in duplex DNA [34].

In our assays of inter-homolog recombination, loss of Top1 had no significant effect on recombination frequency except at the rDNA locus. This result argues that Top1 activity is not a major contributor to the spontaneous recombination events that lead to loss of heterozygosity (LOH). Although the frequency of crossovers between homologs was not affected by Top1 loss, associated gene conversion tracts were significantly longer and more complex that those observed in the corresponding wild-type (WT) strain. This observation suggests that, even though the frequency of initiating lesions is unaffected by Top1, the subsequent steps of recombination are affected. At least *in vitro*, Top1 can overcome a topological stall associated with initial extension of a strand-exchange intermediate [35]. *In vivo*, it is possible that the higher degree of supercoiling expected in the *top1Δ* strain results in longer heteroduplex regions, thereby generating longer gene conversion tracts and more frequent rounds of corrective mismatch repair.

In previous studies, Christman *et al*. [5] found that mutations in either *TOP1* or *TOP2* elevated the frequency of both inter-homolog and inter-sister chromatid recombination in the rDNA. As expected from their results, we found that almost 50% of sub-cultured isolates of the *top1*Δ strain had a terminal region of LOH on chromosome XII with a breakpoint in the rDNA locus (Fig. 6). The rRNA gene cluster may be uniquely sensitive to the loss of Top1 for a variety of reasons. Each of the 9 kb repeats, for example, has two strongly-expressed divergently-transcribed rRNA genes and an origin of replication [36]. Failure to relieve supercoiling may lead to an increased level of stalled replication forks and subsequent DSB formation. In addition, the rDNA cluster is a very large target, representing about 10% of the yeast genome. Destabilization of the rDNA cluster has also been observed in other genetic backgrounds, such as strains with low levels of DNA polymerase α [23], and the rDNA appears to be a natural hotspot for LOH events even in WT strains [37, 38].

In contrast to the unclear expectations of the effects of Top1 loss on recombination, strains with elevated levels of the Top1cc are expected to be hyper-rec. In numerous studies, it has been shown that yeast strains with mutations affecting DSB repair are sensitive to CPT (reviewed by [27, 39]), and there are likely several ways of generating a recombinogenic DNA lesion by CPT. Replication of a DNA molecule with a Top1cc could directly produce a DSB through replication-run-off, or nucleolytic cleavage of a fork regressed because of topological stress could generate a DSB [40]. In addition, some transcription-stimulated mutations have the properties expected from the repair of a Top1cc-associated single-stranded gap [12], which could be processed into a DSB by a nearby nick on the complementary strand.

In our analyses, six hours of CPT treatment elevated the frequency of inter-homolog crossovers on the right arm of chromosome IV and the left arm of chromosome V by 6–8 fold. The *top1-T722A* mutation had a similar quantitative effect on the right arm of chromosome IV, increasing the frequency of crossovers by 5-fold; its effect on chromosome V was not examined. In addition to elevating the rates of recombination on chromosomes IV and V, we observed an elevation in rDNA-associated terminal LOH events on chromosome XII in the *top1-T722A* strain and in the WT strain treated with CPT.

As described previously, the type of gene conversion (3:1, 4:0, hybrid 3:1/4:0, etc.) associated with the crossover in sectored colonies allows us to infer whether the event involved a single broken chromatid (a G2-break) or a pair of sister chromatids broken at the same position (a G1-break). About 75% of the spontaneous events in a wild-type strain are G1 breaks [19]. In the *top1-T722A* mutant or CPT-treated WT strain, only 13% of the crossovers were associated with a G1 break. These results indicate that most Top1cc-associated DNA lesions originate in S or G2, consistent with formation of associated DSBs in the context of DNA replication.

Our recombination assays that involve detection of sectored colonies or microarray-based analysis of genomic DNA primarily detect recombination events between homologs. In particular, recombination between perfectly- or imperfectly aligned sister chromatids does not lead to LOH. Unequal sister-chromatid recombination within a tandem array of genes, however, can lead to a duplication or deletion of repeats that is sometimes detectable by microarrays and this observation provided the initial indication that the tandem array at the *CUP1* locus is destabilized upon accumulation of Top1 cleavage complexes (Fig. 5). We subsequently used Southern blot analysis to more efficiently detect alterations in the number of repeats in the *CUP1* array. This analysis showed that *top1-T722A* mutant and CPT-treated WT strains had highly elevated rates of CNV at the *CUP1* locus.

CNV at *CUP1* could represent either intra- or inter-homolog interactions, but several arguments suggest that most events reflect either inter-sister-chromatid interactions or intra-chromatid events. First, if the copy-number alterations involved inter-homolog recombination, we would have expected to find many sub-cultured isolates with terminal LOH distal to the *CUP1* cluster. No such events were observed. Additionally, Kadyk and Hartwell [41] showed that DNA damage resulting from irradiating G2-synchronized yeast cells was primarily repaired by recombination between sister chromatids rather than inter-homolog recombination.

**Fig 7:**
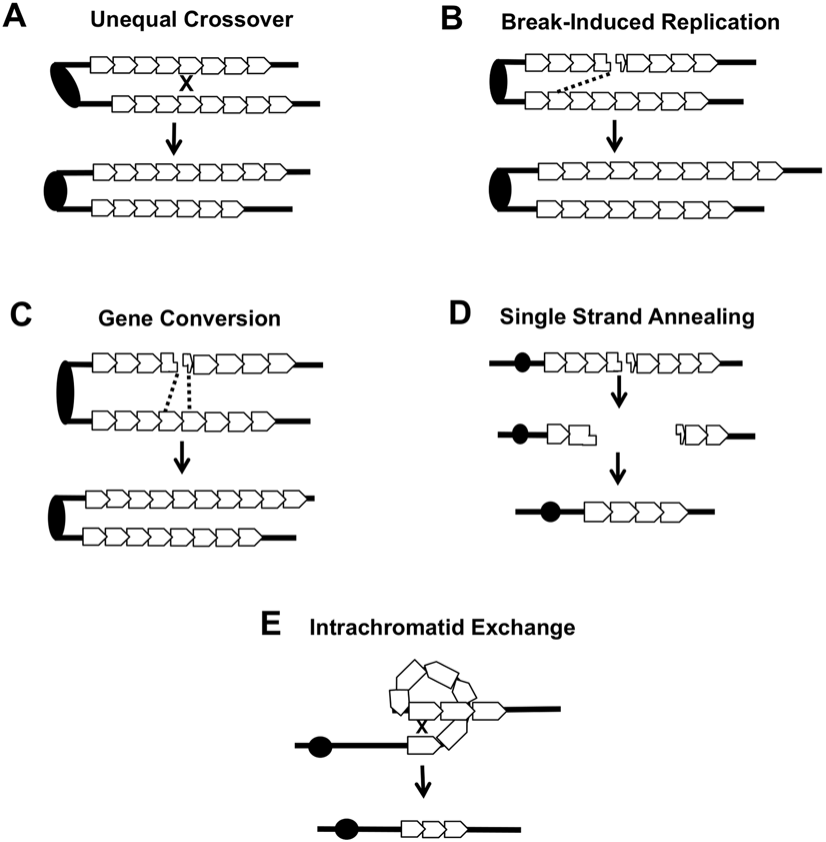
Mechanisms of CNV at the *CUP1* locus. **(A)** An unequal crossover between two misaligned sister chromatids results in one chromatid gaining repeats and the other losing repeats. This event can occur between sister chromatids or homologous chromosomes. **(B)** Break-induced replication (BIR). A DSB can be repaired using the sister chromatid or homolog as a template. Replication to the end of the template chromosome restores the broken chromosome and the template remains unchanged. **(C)** Gene conversion (GC). Repair of a DSB with either the sister chromatid or homologous chromosome by GC results in the repaired chromatid gaining (or losing) copies of the *CUP1* repeat while the donor chromatid remains the same. **(D)** Single strand annealing (SSA). A double strand break (DSB) can be resected to a region of homology and annealing/rejoining of ends leads to a loss of *CUP1* repeats. **(E)** An intrachromatid exchange that involves repeats on the same chromatid results in a loss of multiple *CUP1* repeats.

Several different types of inter-sister or intrachromatid interactions can alter the number of repeats within a tandem array, including unequal crossing-over, break-induced replication (BIR), gene conversion, single-strand annealing (SSA) and intrachromatid “pop-out” recombination that produces an extrachromosomal circle and a shorter array (Fig. 7). Although our current studies cannot distinguish between these mechanisms, the first three are expected to generate both deletions and additions of repeat units, while the latter two produce only deletions. The strong bias we observed for deletions argues that mechanisms producing equal numbers of deletions and additions are likely not the major source of *CUP1* instability. Other studies have indicated that SSA is more common than “pop-out” recombination (summarized by Pâques and Haber [42]), leading us to speculate that SSA is a primary source of CNV at *CUP1*.

In summary, our analysis shows that loss of Top1 and elevated levels of the Top1cc have very different effects on genomic stability, and likely are associated with different types of recombinogenic lesions. Loss of Top1 did not substantially elevate inter-homolog recombination for most of the yeast genome, and had no effect on inter- or intra-sister interactions. An exception to this generalization is that the loss of Top1 resulted in a substantial elevation in inter-homolog recombination in the rDNA, consistent with an earlier study [5]. Although we cannot rule out the possibility that the loss of Top1 increased the rate of recombinogenic DNA lesions in other, single-copy regions of the genome that are repaired almost exclusively by sister-chromatid recombination, we favor the interpretation that loss of Top1 does not produce a high level of lesions except in the rDNA. By contrast, elevated levels of the Top1cc increased the rate of inter-homolog crossover at many sites in the yeast genome, including the rDNA. These complexes also strongly induced CNV formation within the *CUP1* locus. Although there are some differences in the repair of DNA lesions in yeast and mammalian cells [43], it is likely that the recombinogenic effects of Top1 cleavage complexes we have observed in yeast will generate similar types of genome instability in mammalian cells. Indeed, Hashimoto *et al*. [44] and Balestrieri *et al*. [45] reported that CPT treatment of mammalian cells resulted in large deletions and/or rearrangements, but the source and nature of these were not explored. Our findings in yeast are likely relevant to potential downstream effects of using CPT-related compounds in chemotherapy, especially with regard to associated LOH and CNV that could promote tumor progression.

## Materials and Methods

### Strains and plasmids

All experiments were done using diploids formed by crossing derivatives of the haploid strains W303-1A [46] and YJM789 [21]. The resulting diploids were heterozygous for about 55,000 SNPs. The wild-type *TOP1/TOP1* diploids used in the study were JSC25 (*MATa/MATαΔ*::*HYG leu2-3*,*112/LEU2 his3-11*,*15/HIS3 ura3-1/ura3 GAL2/gal2 ade2-1/ade2-1 trp1-1/TRP1 can1-100Δ*::*NAT/CAN1Δ*::*NAT RAD5/RAD5 IV1510386*::*KANMX-can1-100/IVI1510386*::*SUP4-o*; [19]) and PG311 (*MATa/MATαΔ*::*NAT ade2-1/ade2-1 trp1-1/TRP1 ura3-1/URA3 can1-100/can1-Δ*::*SUP4-o gal2/GAL2 ho/ho*::*hisG*; [20]). The constructions of the wild-type diploids JSC25 and PG311 have been described previously [19, 20]. These diploids are isogenic except for changes introduced by transformation; JSC25 has the *SUP4-o* gene located near the right end of chromosome IV, and PG311 has *SUP4-o* located near the left end of chromosome V. The *top1/top1* diploid SLA46.D4 (which has the *TOP1*-containing plasmid pWJ1491) is a derivative of SLA43D generated by replacing the *MATα* gene with the *HYG* marker. This replacement was done using a PCR fragment amplified from a *MATαΔ*::*HYG* strain (SLA46.19) using the primers Malpha::URA3F(big) (5′-AATCGTCCTGTCCCATTACG) and Malpha::URA3R(big) (5′-TTGGAAACACCAAGGGAGAG). SLA43D was constructed by mating the haploids SLA36.A and SLA42.5. SLA36.A was constructed in several steps. First, W1588-4C, a *RAD5* derivative of W303-1A [47], was transformed with a *top1*Δ-containing PCR fragment generated by amplifying the *NAT*-containing plasmid pAG25 [48] with primers top1-NAT F (5′-TCTCTGTTACTCTAATTACCTGAGTCCTATTCTTATAGTATTAAAACAGCCGTACGCTGCAGGTCGAC) and top1-NAT R (5′-ACTTGATGCGTGAATGTATTTGCTTCTCCCCTATGCTGCGTTTCTTTGCGATCGATGAATTCGAGCTCG). The resulting *top1Δ* strain (SLA24.5) was transformed with a PCR fragment generated by amplifying the plasmid pFA6a [49] with primers 1510336 KANMX (5′-CCTATTTTTCATACGTTATGCACTTCATTCTTCTTGTCGGTTTGATAACAACGCTGCAGGTCGAC) and 1510435 KANMX (5′-GGTATGGCTTCTGCCGGGCTAACGTTCAAATTAAAGGAACTAGATTCTGCATCGATGAATTCGAGCTCG). The resulting strain (SLA30) had an insertion of the *KANMX* cassette near the right end of chromosome IV. The strain SLA30 was transformed with the *TOP1*-containing plasmid pWJ1491 (described below) to generate the haploid SLA36.A.

SLA42.5, the other haploid parent of SLA46.D4, is isogenic with YJM789 [21] except for changes introduced by transformation. To generate this strain, haploid PSL4 [20] was transformed with a PCR fragment produced by amplifying genomic DNA of JSC21 [19] with primers IV:SUP4-o Wide F (5′-AACCGCCGGAAGAAGTTTGG) and IV:SUP4-o Wide R (5′-AGTTGTAATGGTTCTACCTAGCAAAGG). The resulting strain (SLA35.9) had an insertion of *SUP4-o* near the right end of chromosome IV. SLA42.5, the *top1Δ*::*NAT* derivative of SLA35.9, was constructed by the same method as described above for SLA24.5.

In different experiments, SLA46.D4 was transformed with pRS416 (*CEN-* and *URA3*-containing control plasmid; [50]), pWJ1490 (pRS416 containing the *pCUP1-top1-T772A* gene), or pWJ1491 (pRS316 containing the *pCUP1-TOP1* gene). The control vector pRS416 was described previously [50]. The plasmids pWJ1490 and pWJ1491 were derived from the plasmids pWJ1440 and pWJ1441 [29], respectively, and were constructed by R. Reid and R. Rothstein (Columbia University). The plasmid pWJ1440 was treated with *Eag*I, and the fragment containing the *pCUP1-top1-T722A* fusion gene was inserted into the *Eag*I site of pRS416 to make pWJ1490. The same approach was used to insert the *pCUP1-TOP1* gene derived from pWJ1441 into *Eag*I-treated pRS416 to construct pWJ1491.

### Colony sector analysis

For CPT sectoring assays, cells were grown non-selectively in YPD (1% yeast extract, 2% Bacto-peptone, 2% dextrose; 2% agar for plates) with only 10μg/ml adenine hemisulfate [18–20, 22] to saturation, diluted to 3x10^5^ cells/mL in YPD medium containing either 500μM CPT (Sigma C9911, 20mg/ml stock dissolved in DMSO) or DMSO (untreated control) and grown at 30°C for 6 hours. After CPT treatment, cells were washed with H_2_O and appropriate dilutions were plated on synthetic complete dextrose medium containing a reduced amount of adenine hemisulfate (10μg/ml) and lacking arginine with (SD/low Ade-Arg) to determine total cell number and screen for sectors. In the case of PG311, cells were plated on SD/low Ade-Arg plates that contained 240μg/ml canavanine to screen for sectors; a higher concentration of canavanine was used to reduce background growth.

To measure sectoring in a *top1*Δ background, SLA46.D4 was streaked on YPD plates and then replica plated onto synthetic complete dextrose medium lacking uracil (SD-Ura) to identify colonies that had lost the complementing *TOP1-URA* plasmid (pWJ1491). To examine the effect of the *top1-T722A* allele on sectoring, appropriate plasmids were introduced by lithium acetate transformation and transformants were selected on SD-Ura plates [12]. Individual Ura^+^ colonies were resuspended in water and an appropriate dilution plated on SD/low Ade-Arg-Ura plates to determine total cell number and to screen for sectors. Following purification, presence of the *SUP4-o* marker in the white portion of the sector was confirmed by patching onto SD-Ade medium; presence of the appropriate drug resistant marker in the red sector was confirmed by patching onto YPD plates containing Kanamycin (for JSC25; Fig. 1) or Hygromycin (for PG311; S1 Fig). Only verified sectors were used to calculate sectoring rates.

### Sub-culturing experiments

To measure the effects of CPT on genome stability, we streaked JSC25 or PG311 to yield single colonies on YPD plates, YPD plates containing 500μM CPT + DMSO, or YPD plates containing an equivalent amount of DMSO. Plates were incubated at 30°C for two days and a single colony was then streaked again onto the same type of medium. The re-streaking of a single colony was repeated 10 times, with each re-streaking corresponding to one sub-culture. Following transformation of a *TOP1*-containing, *top1-T722A*-containing or control plasmid into SLA46.D4, selected colonies were sub-cultured as described above on SD-Ura plates in order to maintain the plasmids.

### Analysis of LOH using microarrays

Oligonucleotide-containing DNA microarrays that contained oligonucleotides were perfectly matched to either W303-1A-specific SNPs or YJM789-specific SNPs; similar arrays were used previously by Gresham *et al*. [51]. Two types of microarrays were used: one that was specific for SNPs located on the right arm of chromosome IV [19] and one that could be used to assay LOH throughout the genome [18].

Using the methods employed previously [19, 52], we examined correlations between the breakpoints of these events and various chromosome elements such as centromeres, tRNA genes, and retrotransposons.

### Genome-element association analysis for COs on right arm of chromosome IV

To determine whether particular genome features were over- or under-represented at CO breakpoints, we first delineated a window for each CO as the region most likely to have contained the recombinogenic DNA break. In the case of the *top1Δ* sectors analyzed, the windows included from the last heterozygous SNP to the first homozygous SNP. The windows were summed (2.00 x 10^6^ kb total) and then divided by the total amount of the genome that was screened for COs (1.1x10^6^ kb of chromosome IV in each of 88 sectors, or 9.5x10^7^ kb total). This calculation yielded 2.12 x10^-2^ as the fraction of the total kb examined that is contained within the windows. For each genome feature we determined how many total features were detectable within the 9.5x10^7^ kb total region screened, then multiplied that by the fraction of the region found within the windows (2.12x10^-2^) to yield an expected number of features. For example, there are 28 ARS elements within the 1.1x10^6^ kb of sequence screened for COs on the right arm of chromosome IV and, therefore, 2464 ARS elements total in the 88 sectors. Of the 2464 ARS elements, 52 (2464 times 2.12x10^-2^) are predicted to fall within our CO-associated windows. We counted the number of each feature that overlapped with the CO-associated windows to generate the observed number. Chi-square analysis was then used to compare the expected and observed numbers for each genome feature. For Ty elements we used midpoint coordinates rather than the full element, as their large size made them more likely to overlap the windows and be over-represented. The same methods were used to delineate genome associations from the 20 CPT-treated and 20 *top1-T722A* expressing sectors.

### Analysis of a *Spe*I polymorphism to detect RCOs initiating in the rDNA locus

A region ~21 kb downstream of the rDNA locus on Chromosome XII was PCR amplified from the genomic DNA or sub-cultured colonies. A PCR product of about 750 bp was generated using primers ChrXIIF490730 (5′-CTGATGAGTTCTGCATCTGTCC) and ChrXIIR491473 (5′-TCCGTTACCATTGCATACAGAA-3’). Within this fragment, there is a *Spe*I restriction site specific to the YJM789 allele. Digestion of the PCR product with *Spe*I results in two fragments of about 500 and 250 bp when the YJM789 allele is present, and a single 750 bp fragment when the W303-1A allele is present. *Spe*I digests were analyzed on a 1% agarose gel to determine whether the diploid strain remained heterozygous or became homozygous for relevant SNP during sub-culturing.

### Southern analysis of *CUP1* tandem arrays

Genomic DNA was isolated from 5 ml of saturated YPD cultures using a modified standard isolation procedure (http://jinks-robertsonlab.duhs.duke.edu/protocols/yeast_prep.html) or was extracted in agarose plugs as described by Lobachev *et al*. [53]. If DNA plugs were used, the plugs were equilibrated in CutSmart Restriction Digestion Buffer before overnight digestion with *Eco*R. Digested samples were examined by electrophoresis on 1% agarose using the BioRad CHEF Mapper XA System. The switching interval was optimized for DNA molecules in the 10–60 kb range. Following transfer of the separated DNA fragments to a nitrocellulose membrane, the membrane was hybridized to a DIG-labeled *CUP1* probe at 42°C for at least 16 hours. To obtain the probe, we PCR-amplified a 1 kb segment of the *CUP1* repeat (including the entire *CUP1* gene) from genomic DNA of an isogenic derivative of the strain SJR282 [54] using primers CUP1-amp3 and CUP1-amp5-2 [30]. The PCR product was then labeled with digoxigenin-UTP (DIG) using the Roche Diagonostics DIG-High Prime DNA Labeling and Detection Starter Kit II (Roche 11585614910). Hybridization of the probe to the filter was detected with the CSPD [disodium 3-(4-methoxyspiro {1,2-dioxetane-3,2’-(5’-chloro)tricycle [3.3.1.1^3,7^]decan}-4-yl)phenyl phosphate] chemiluminescent alkaline phosphatase substrate. Alterations in the number of *CUP1* repeats per array was detected by comparing the sizes of *CUP1*-hybridizing fragments to those in the starting strain. When multiple bands were observed, our conclusions were based on the two strongest bands. To estimate the number of *CUP1* repeats lost or gained, we plotted the molecular size in kb versus the distance migrated in millimeters using Bioline Hyperladder VI or BioRad CHEF DNA Size Standard as standards. We then generated a trend line to estimate the size of the *CUP1* fragment based on the distance migrated.

### Other genetic and physical methods

Standard methods were used for mating, transformation, and media preparation [55].

### Statistical analysis

Depending on the experiment, statistical comparisons were done using the chi-square test, the Fisher exact test, or the non-parametric Mann-Whitney test. These tests were done using the VassarStats Website (http://vassarstats.net). To determine 95% confidence limits on the proportions of sectored colonies in Fig. 4, we used Method 3 described by Newcombe [56].

## Supporting Information

S1 Fig PG311, Chr V diploid strain. Chromatids in red are derived from W303-1A and contain a *can1-100* ochre mutation with a closely-linked Hygromycin resistance cassette (HYG^R^, yellow box) cassette on the left arm of chromosome V. Chromatids in black are derived from YJM789 and contain a *SUP4-o* marker on the left arm of chromosome V. In addition, the diploid strain is homozygous for the *ade2-1* mutation. In the event a reciprocal crossover occurs that leads to loss of heterozygosity (LOH), a red/white sectored Can^R^ colony will form after the daughter cells segregate.
(TIFF)

S1 Table SGD coordinates for heterozygous and homozygous transitions on chromosome IV in *top1Δ* red/white sectors. ^1^The event class of each sector is listed, using classification in a previous study [19]. ^2^Transition label (lowercase letters) represent each transition from heterozygosity to homozygosity in a conversion tract. ^3^Markers flanking transitions are the SGD coordinates of SNPs located on each side of the transition. All reported coordinates are based on Feb. 2010 SGD coordinates and may be different from those currently in SGD.
(DOCX)

S2 Table Conversion tracts associated with RCOs in *top1Δ* red/white sectors. The top line represents the red side of the indicated sector and the bottom line represents the white side of the sector. Green, red and black represent heterozygosity for SNPs, homozygosity for W303-1A SNPs, and homozygosity for YJM789 SNPs, respectively. The lowercase letters located above and below the lines mark transitions from heterozygosity to homozygosity and are correlated with transition labels shown in S1 Table. Blue boxes enclose the regions that are likely to be associated with DSB formation; for details on the event classification of sectors, please refer to St. Charles *et al*.*es* [19]. Novel class of sectors (N) are sectors that have not been observed in previous studies. The lengths of conversion tracts are not drawn to scale. One asterisk (*) and two asterisks (**) indicate a G1 and G2 DSB, respectively.
(PDF)

S3 Table SGD coordinates for heterozygous and homozygous transitions on chromosome IV in CPT treated red/white sectors. See S1 Table legend for details.
(DOCX)

S4 Table Conversion tracts associated with RCOs in CPT treated red/white sectors. See S2 Table legend for details.
(PDF)

S5 Table SGD coordinates for heterozygous and homozygous transitions on chromosome IV in Top1-T722A red/white sectors. See S1 Table legend for details.
(DOCX)

S6 Table Conversion tracts associated with RCOs in Top1-T722A red/white sectors. See S2 Table legend for details.
(PDF)

S7 Table SGD coordinates for events detected in sub-cultured clones. For each detected event, an event class (*) is listed that is correlated with S8 Table. ** Markers flanking transitions: SGD coordinates of SNPs located on each side of the transition. All reported coordinates are based on SGD coordinates from Feb. 2010 and may be different from those posted in SGD.
(DOCX)

S8 Table Chromosome depictions of events in clones sub-cultured 10 times. The colored line represents a chromosome. Green, red and black represent heterozygosity for SNPs, homozygosity for W303-1A SNPs, and homozygosity for YJM789 SNPs, respectively. The lengths of chromosomes are not drawn to scale.
(PDF)

S9 Table *CUP1* Southern data for sub-cultured clones. Southern analysis data for the behavior of the *CUP1* arrays in W303-1A and YJM789 are listed for all sub-cultured 1 and 10 clones. NC, # in blue and # in red represent no detectable change, estimated number of repeats lost, and estimated number of repeats gained, respectively. For CPT-treated and Top1-T722A sub-cultured clones, a column for instability is included, signifying that more than two bands were detected by Southern analysis. It should be noted that instability was only detected in these clones and not in untreated (WT) or *top1Δ* clones.
(DOCX)

S10 Table Reciprocal crossovers initiated within the rDNA locus after 10 sub-cultures. The total number of sub-cultured clones analyzed is in the rightmost column. The column labeled “Both” indicates the number of these clones that were heterozygous for the SNP located distal to the rDNA locus. We also show the number of sub-clones that were homozygous for the W303-1A- and YJM789-derived SNPs.
(DOCX)
