# Supplementary figures and images for: Genome-Destabilizing Effects Associated with Top1 Loss or Accumulation of Top1 Cleavage Complexes in Yeast
Source: PLoS Genet. 2015 Apr 1;11(4):e1005098. doi: 10.1371/journal.pgen.1005098 (PMC4382028; doi:10.1371/journal.pgen.1005098)

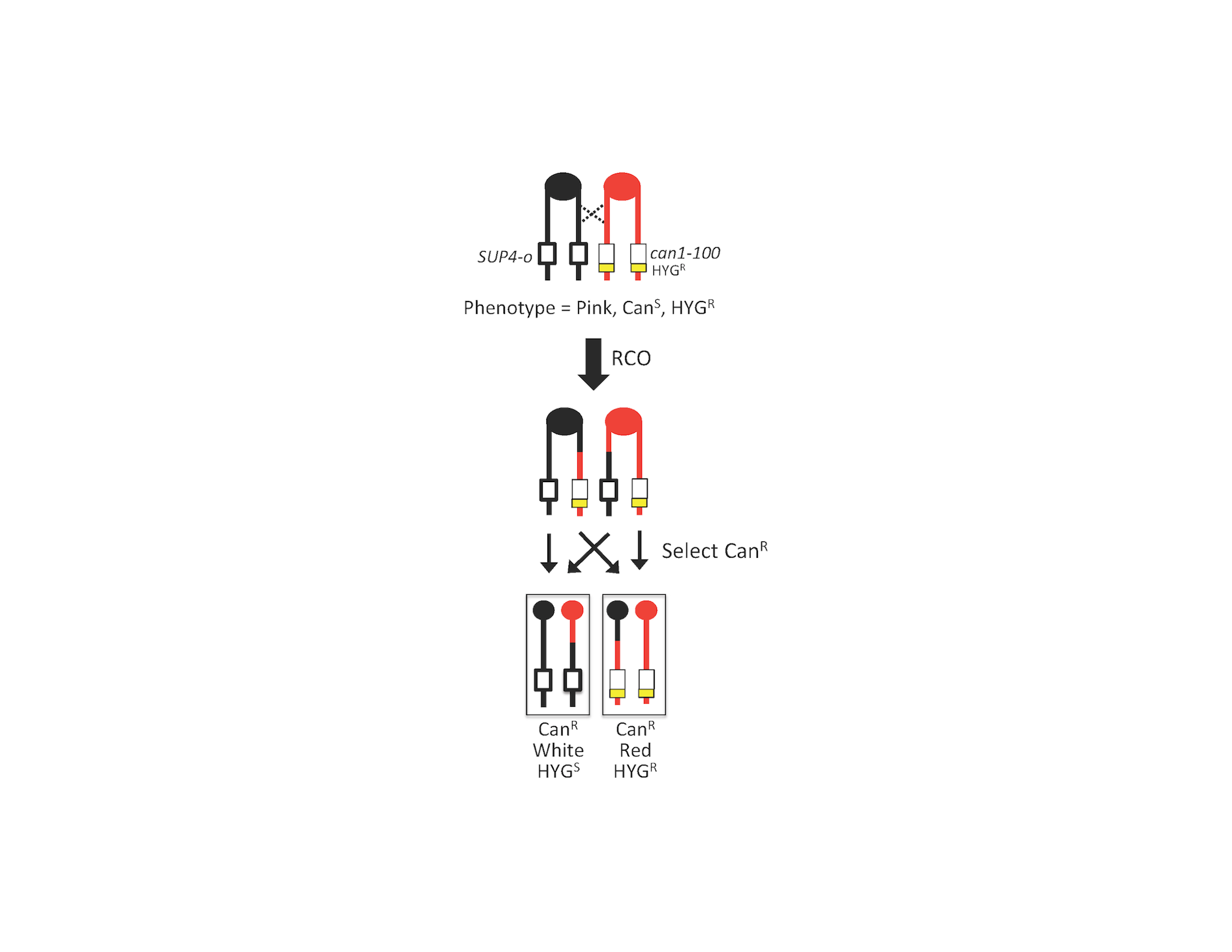

Supplement: S1 Fig — Chromatids in red are derived from W303-1A and contain a can1-100 ochre mutation with a closely-linked Hygromycin resistance cassette (HYGR, yellow box) cassette on the left arm of chromosome V. Chromatids in black are derived from YJM789 and contain a SUP4-o marker on the left arm of chromosome V. In addition, the diploid strain is homozygous for the ade2-1 mutation. In the event a reciprocal crossover occurs that leads to loss of heterozygosity (LOH), a red/white sectored CanR colony will form after the daughter cells segregate. (TIFF) [file pgen.1005098.s001.tiff]
