# Supplementary material for: Genome-Destabilizing Effects Associated with Top1 Loss or Accumulation of Top1 Cleavage Complexes in Yeast
Source: PLoS Genet. 2015 Apr 1;11(4):e1005098. doi: 10.1371/journal.pgen.1005098 (PMC4382028; doi:10.1371/journal.pgen.1005098)
Supplement: S1 Table — 1The event class of each sector is listed, using classification in a previous study [19]. 2Transition label (lowercase letters) represent each transition from heterozygosity to homozygosity in a conversion tract. 3Markers flanking transitions are the SGD coordinates of SNPs located on each side of the transition. All reported coordinates are based on Feb. 2010 SGD coordinates and may be different from those currently in SGD. (DOCX) [file pgen.1005098.s002.docx]

**S1 Table. SGD coordinates for heterozygous and homozygous transitions on** **chromosome IV in *top1∆* red/white sectors.**

| Sector | Event Class^1^ | Transition Label^2^ | Markers flanking transitions^3^ | | |
| --- | --- | --- | --- | --- | --- |
|  |  |  | Left | Right | |
| 4RW | B1 | a | 1075942 | | 1089446 |
|  |  | b | 1089446 | | 1111969 |
| 5RW | D6 | a | 471247 | | 477827 |
|  |  | b | 485657 | | 491787 |
|  |  | c | 491787 | | 495183 |
|  |  | d | 505247 | | 505383 |
| 7RW | D6 | a | 1438183 | | 1441463 |
|  |  | b | 1446463 | | 1446859 |
|  |  | c | 1472998 | | 1473819 |
|  |  | d | 1476496 | | 1477097 |
| 9RW | C2 | a | 889108 | | 892648 |
|  |  | b | 895029 | | 909432 |
| 13RW | D3 | a | 1181603 | | 1181992 |
|  |  | b | 1186611 | | 1187255 |
|  |  | c | 1190182 | | 1216161 |
| 15RW | B2 | a | 722446 | | 724557 |
|  |  | b | 729016 | | 736646 |
| 17BRW | B1 | a | 600750 | | 601671 |
|  |  | b | 611081 | | 614724 |
| 22RW | A | a | 1375238 | | 1379772 |
| 23RW | N1 | a | 1296369 | | 1297053 |
|  |  | b | 1299154 | | 1299579 |
|  |  | c | 1299579 | | 1299788 |
|  |  | d | 1299788 | | 1300026 |
|  |  | e | 1300815 | | 1301076 |
|  |  | f | 1305574 | | 1305759 |
|  |  | g | 1305759 | | 1306176 |
|  |  | h | 1307608 | | 1308659 |
|  |  | i | 1312507 | | 1313440 |
|  |  | j | 1314892 | | 1315266 |
|  |  | k | 1315266 | | 1315889 |
| 24RW | C2 | a | 1392166 | | 1394070 |
|  |  | b | 1394070 | | 1397989 |
| 28RW | E26 | a | 1374198 | | 1379772 |
|  |  | b | 1385030 | | 1386901 |
|  |  | c | 1387294 | | 1391336 |
|  |  | d | 1394070 | | 1397989 |
| 29RW | A | a | 1111969 | | 1142607 |
| 39RW | B2 | a | 777624 | | 778109 |
|  |  | b | 796161 | | 802110 |
| 40RW | A | a | 1111969 | | 1142607 |
| 41RW | A | a | 1079615 | | 1089446 |
| 43RW | D4 | a | 1362046 | | 1364446 |
|  |  | b | 1367557 | | 1368003 |
|  |  | c | 1371117 | | 1371846 |
| 44RW | N2 | a | 895029 | | 907253 |
|  |  | b | 907253 | | 911910 |
|  |  | c | 911910 | | 912481 |
|  |  | d | 914889 | | 917449 |
|  |  | e | 924604 | | 926722 |
| 48RW | N3 | a | 471247 | | 477827 |
|  |  | b | 477827 | | 482169 |
|  |  | c | 485707 | | 491787 |
|  |  | d | 491787 | | 495183 |
|  |  | e | 495183 | | 496923 |
|  |  | f | 507960 | | 509085 |
| 49RW | N4 | a | 561291 | | 562957 |
|  |  | b | 565455 | | 565640 |
|  |  | c | 566713 | | 567792 |
|  |  | d | 574605 | | 574954 |
|  |  | e | 575577 | | 575760 |
|  |  | f | 576294 | | 578438 |
|  |  | g | 578438 | | 578634 |
|  |  | h | 579229 | | 579742 |
| 50RW | N5 | a | 1221462 | | 1231783 |
|  |  | b | 1235569 | | 1238591 |
|  |  | c | 1239335 | | 1239971 |
|  |  | d | 1240118 | | 1246214 |
|  |  | e | 1250175 | | 1250503 |
|  |  | f | 1252616 | | 1253315 |
| 51RW | B2 | a | 611611 | | 614724 |
|  |  | b | 619170 | | 620076 |
| 52RW | N6 | a | 768023 | | 770269 |
|  |  | b | 771197 | | 772684 |
|  |  | c | 774274 | | 774716 |
|  |  | d | 778449 | | 780003 |
|  |  | e | 782849 | | 784045 |
|  |  | f | 784045 | | 785144 |
|  |  | g | 785867 | | 785910 |
|  |  | h | 786014 | | 786493 |
| 58RW | B2 | a | 820458 | | 820996 |
|  |  | b | 830107 | | 830605 |
| 61RW | N7 | a | 844035 | | 845553 |
|  |  | b | 845725 | | 845945 |
|  |  | c | 845945 | | 846463 |
|  |  | d | 849782 | | 850150 |
|  |  | e | 851194 | | 852302 |
| 69RW | N8 | a | 1461572 | | 1461942 |
|  |  | b | 1468543 | | 1468701 |
|  |  | c | 1468701 | | 1468820 |
|  |  | d | 1469944 | | 1470362 |
|  |  | e | 1471510 | | 1471808 |
|  |  | f | 1472155 | | 1472317 |
|  |  | g | 1472810 | | 1472998 |
|  |  | h | 1473297 | | 1473612 |
|  |  | i | 1480252 | | 1483848 |
|  |  | j | 1486159 | | 1486789 |
|  |  | k | 1486789 | | 1487856 |
| 71RW | E5 | a | 890618 | | 892648 |
|  |  | b | 895029 | | 901094 |
|  |  | c | 901094 | | 907253 |
| 81RW | D6 | a | 730063 | | 730326 |
|  |  | b | 733517 | | 735346 |
|  |  | c | 736646 | | 737994 |
|  |  | d | 737994 | | 739042 |
| 82RW | N9 | a | 1495013 | | 1495494 |
|  |  | b | 1499309 | | 1500032 |
|  |  | c | 1501340 | | 1501434 |
|  |  | d | 1501654 | | 1501862 |
|  |  | e | 1501917 | | 1502531 |
| 83RW | B1 | a | 625236 | | 628588 |
|  |  | b | 643241 | | 643421 |
| 84RW | B1 | a | 1392166 | | 1394070 |
|  |  | b | 1398890 | | 1399604 |
| 85RW | A | a | 1067771 | | 1069072 |
| 86RW | N10 | a | 730720 | | 731914 |
|  |  | b | 732258 | | 733517 |
|  |  | c | 733517 | | 735346 |
| 87RW | N11 | a | 500579 | | 500992 |
|  |  | b | 503234 | | 503397 |
|  |  | c | 505037 | | 505120 |
|  |  | d | 505120 | | 505159 |
|  |  | e | 509817 | | 512538 |
|  |  | f | 512801 | | 521609 |
|  |  | g | 521609 | | 524793 |
| 88RW | E3 | a | 1075942 | | 1077836 |
|  |  | b | 1079615 | | 1089446 |
| 89RW | N12 | a | 747021 | | 747660 |
|  |  | b | 752071 | | 753192 |
|  |  | c | 760133 | | 761321 |
|  |  | d | 766033 | | 766674 |
|  |  | e | 770717 | | 770845 |
|  |  | f | 770845 | | 771197 |
|  |  | g | 772684 | | 773514 |
| 90RW | N13 | a | 845377 | | 845553 |
|  |  | b | 862566 | | 863830 |
|  |  | c | 868904 | | 889108 |
|  |  | d | 980838 | | 992782 |
| 91RW | B1 | a | 1013909 | | 1015206 |
|  |  | b | 1015206 | | 1017595 |
| 92RW | D5 | a | 579993 | | 580364 |
|  |  | b | 583752 | | 584806 |
|  |  | c | 584836 | | 585304 |
|  |  | d | 592210 | | 593108 |
| 93RW | B2 | a | 1111969 | | 1142607 |
|  |  | b | 1156773 | | 1161545 |
| 94RW | D6 | a | 960043 | | 961833 |
|  |  | b | 965740 | | 968451 |
|  |  | c | 993113 | | 993284 |
|  |  | d | 1008881 | | 1009116 |
| 95RW | N14 | a | 1475382 | | 1476496 |
|  |  | b | 1477097 | | 1479510 |
|  |  | c | 1492290 | | 1492805 |
|  |  | d | 1493232 | | 1493451 |
|  |  | e | 1493508 | | 1493713 |
| 96RW | B2 | a | 1003836 | | 1005641 |
|  |  | b | 1009543 | | 1009894 |
| 98RW | B1 | a | 484013 | | 491787 |
|  |  | b | 495183 | | 497451 |
| 99RW | D5 | a | 755087 | | 759928 |
|  |  | b | 766993 | | 767421 |
|  |  | c | 768023 | | 770269 |
|  |  | d | 770845 | | 771197 |
| 100RW | D2 | a | 961922 | | 962530 |
|  |  | b | 994215 | | 995622 |
|  |  | c | 1010105 | | 1013909 |
| 103RW | N15 | a | 690253 | | 690489 |
|  |  | b | 692783 | | 693056 |
|  |  | c | 694817 | | 695355 |
|  |  | d | 696269 | | 696472 |
|  |  | e | 696542 | | 697181 |
|  |  | f | 698494 | | 699188 |
| 104RW | E2 | a | 993113 | | 993175 |
|  |  | b | 993347 | | 994109 |
|  |  | c | 1000365 | | 1001602 |
|  |  | d | 1017595 | | 1036052 |
| 107RW | N16 | a | 794806 | | 796086 |
|  |  | b | 796161 | | 802539 |
|  |  | c | 806034 | | 806160 |
|  |  | d | 806160 | | 806219 |
| 108RW | D6 | a | 628588 | | 629711 |
|  |  | b | 632639 | | 633984 |
|  |  | c | 637196 | | 638827 |
|  |  | d | 641493 | | 641771 |
| 109RW | A | a | 1489226 | | 1491125 |
| 110RW | N16 | a | 952255 | | 953272 |
|  |  | b | 953272 | | 955816 |
|  |  | c | 960710 | | 961922 |
|  |  | d | 969944 | | 970231 |
| 111RW | B2 | a | 1374246 | | 1379772 |
|  |  | b | 1379772 | | 1385030 |
| 113RW | N17 | a | 735346 | | 735603 |
|  |  | b | 736646 | | 737994 |
|  |  | c | 755087 | | 755533 |
|  |  | d | 755533 | | 759928 |
| 128RW | B2 | a | 1312482 | | 1313440 |
|  |  | b | 1330633 | | 1330866 |
| 132RW | N18 | a | 1426190 | | 1427147 |
|  |  | b | 1429103 | | 1430099 |
|  |  | c | 1430473 | | 1432266 |
| 134RW | N19 | a | 1334123 | | 1336486 |
|  |  | b | 1336931 | | 1337173 |
|  |  | c | 1337217 | | 1337696 |
|  |  | d | 1337898 | | 1338243 |
| 136RW | N20 | a | 498729 | | 498933 |
|  |  | b | 499633 | | 500500 |
|  |  | c | 500500 | | 500992 |
|  |  | d | 500992 | | 502459 |
| 138RW | N21 | a | 1089446 | | 1111969 |
|  |  | b | 1153158 | | 1156033 |
|  |  | c | 1157254 | | 1161545 |
|  |  | d | 1161545 | | 1171413 |
|  |  | e | 1190182 | | 1216211 |
| 139RW | B2 | a | 857606 | | 862566 |
|  |  | b | 862566 | | 865277 |
| 141RW | B1 | a | 747660 | | 749244 |
|  |  | b | 755087 | | 759928 |
| 142RW | B1 | a | 1327684 | | 1328086 |
|  |  | b | 1346317 | | 1346791 |
| 143RW | B2 | a | 571796 | | 572478 |
|  |  | b | 581508 | | 581984 |
| 144RW | D1 | a | 594021 | | 597003 |
|  |  | b | 598715 | | 599224 |
|  |  | c | 599224 | | 601671 |
| 146RW | N22 | a | 667844 | | 668462 |
|  |  | b | 670310 | | 670622 |
|  |  | c | 679982 | | 680109 |
|  |  | d | 681650 | | 681904 |
| 148RW | B1 | a | 755533 | | 760133 |
|  |  | b | 773560 | | 773793 |
| 149RW | N23 | a | 889108 | | 892648 |
|  |  | b | 894438 | | 895029 |
|  |  | c | 895029 | | 901094 |
|  |  | d | 907253 | | 911910 |
| 150RW | A | a | 1089446 | | 1142607 |
| 151RW | B1 | a | 1071758 | | 1142607 |
|  |  | b | 1147563 | | 1147830 |
| 152RW | N24 | a | 1235569 | | 1238591 |
|  |  | b | 1255161 | | 1255275 |
|  |  | c | 1255275 | | 1255326 |
|  |  | d | 1255964 | | 1256206 |
|  |  | e | 1256631 | | 1257078 |
|  |  | f | 1265332 | | 1266551 |
|  |  | g | 1266551 | | 1267763 |
| 154RW | N25 | a | 974674 | | 974975 |
|  |  | b | 974975 | | 975111 |
|  |  | c | 975687 | | 977414 |
|  |  | d | 977752 | | 978557 |
|  |  | e | 979514 | | 979777 |
|  |  | f | 993347 | | 994215 |
|  |  | g | 1000365 | | 1001602 |
|  |  | h | 1004465 | | 1005641 |
| 156RW | B2 | a | 459452 | | 469765 |
|  |  | b | 469765 | | 470421 |
| 157RW | B2 | a | 892750 | | 895029 |
|  |  | b | 895029 | | 911910 |
| 159RW | N26 | a | 668847 | | 670283 |
|  |  | b | 670283 | | 671011 |
|  |  | c | 672337 | | 672738 |
|  |  | d | 677821 | | 678096 |
|  |  | e | 678096 | | 679246 |
| 160RW | A | a | 781765 | | 784045 |
|  |  | b | 784045 | | 784283 |
| 161RW | B1 | a | 661148 | | 663847 |
|  |  | b | 667844 | | 668462 |
| 162RW | A | a | 1231783 | | 1235569 |
| 164RW | N27 | a | 485657 | | 495183 |
|  |  | b | 497451 | | 497515 |
|  |  | c | 498123 | | 498293 |
|  |  | d | 498293 | | 498729 |
|  |  | e | 512538 | | 512756 |
|  |  | f | 512756 | | 512801 |
| 165RW | A | a | 598715 | | 599224 |
| 166RW | N35 | a | 507594 | | 509085 |
|  |  | b | 509392 | | 509716 |
|  |  | c | 512538 | | 512801 |
|  |  | d | 512801 | | 521609 |
|  |  | e | 526934 | | 539130 |
|  |  | f | 551574 | | 551730 |
|  |  | g | 559223 | | 560565 |
| 167RW | N28 | a | 1431782 | | 1432510 |
|  |  | b | 1433911 | | 1438183 |
|  |  | c | 1441931 | | 1443274 |
|  |  | d | 1443274 | | 1445622 |
| 168RW | N29 | a | 1221462 | | 1235569 |
|  |  | b | 1240156 | | 1246274 |
|  |  | c | 1247369 | | 1248070 |
|  |  | d | 1256631 | | 1257078 |
| 169RW | N30 | a | 1379772 | | 1385030 |
|  |  | b | 1398890 | | 1399604 |
|  |  | c | 1399604 | | 1399918 |
|  |  | d | 1410143 | | 1411220 |
|  |  | e | 1418918 | | 1421111 |
|  |  | f | 1423586 | | 1424806 |
| 171RW | N31 | a | 1318359 | | 1318731 |
|  |  | b | 1320551 | | 1322123 |
|  |  | c | 1322123 | | 1322287 |
|  |  | d | 1322287 | | 1323849 |
|  |  | e | 1328086 | | 1330102 |
|  |  | f | 1330102 | | 1330538 |
|  |  | g | 1339571 | | 1340268 |
|  |  | h | 1340825 | | 1341268 |
| 172RW | N32 | a | 1438183 | | 1441741 |
|  |  | b | 1446405 | | 1446859 |
|  |  | c | 1449070 | | 1450072 |
|  |  | d | 1453193 | | 1455114 |
| 173RW | E11 | a | 1470765 | | 1471982 |
|  |  | b | 1472155 | | 1472810 |
|  |  | c | 1474120 | | 1474375 |
|  |  | d | 1480252 | | 1483848 |
| 174RW | D5 | a | 1231783 | | 1235569 |
|  |  | b | 1235569 | | 1238628 |
|  |  | c | 1238628 | | 1240303 |
|  |  | d | 1240303 | | 1246691 |
| 175RW | N33 | a | 655439 | | 658422 |
|  |  | b | 658422 | | 659898 |
|  |  | c | 661123 | | 663847 |
|  |  | d | 667425 | | 668847 |
|  |  | e | 670283 | | 671011 |
|  |  | f | 673223 | | 673947 |
| 176RW | N34 | a | 1330102 | | 1330358 |
|  |  | b | 1330358 | | 1330538 |
|  |  | c | 1354259 | | 1365816 |
|  |  | d | 1369831 | | 1370985 |
|  |  | e | 1374333 | | 1379772 |

^1^ Event Class: Classes of events are defined in Table S2.

^2^ Transition Label: These lower case letters reflect the transition from heterozygous to homozygous regions as shown in Table S2.

^3^ Markers flanking transition: SGD coordinates of SNPs located on each side of the transition.
