# Supplementary material for: Genome-Destabilizing Effects Associated with Top1 Loss or Accumulation of Top1 Cleavage Complexes in Yeast
Source: PLoS Genet. 2015 Apr 1;11(4):e1005098. doi: 10.1371/journal.pgen.1005098 (PMC4382028; doi:10.1371/journal.pgen.1005098)
Supplement: S2 Table — The top line represents the red side of the indicated sector and the bottom line represents the white side of the sector. Green, red and black represent heterozygosity for SNPs, homozygosity for W303-1A SNPs, and homozygosity for YJM789 SNPs, respectively. The lowercase letters located above and below the lines mark transitions from heterozygosity to homozygosity and are correlated with transition labels shown in S1 Table. Blue boxes enclose the regions that are likely to be associated with DSB formation; for details on the event classification of sectors, please refer to St. Charles et al.es [19]. Novel class of sectors (N) are sectors that have not been observed in previous studies. The lengths of conversion tracts are not drawn to scale. One asterisk (*) and two asterisks (**) indicate a G1 and G2 DSB, respectively. (PDF) [file pgen.1005098.s003.pdf]

S2 Table. Conversion tracts associated with reciprocal crossovers in *top1*∆ red/white sectors

Class	Number observed	
**A**	**10**	**Simple crossover, no conversion tract**
		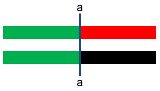
**B**	**23**	**3:1 conversion tract**
B1**	11	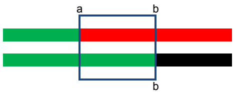
B2**	12	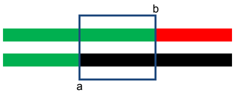
**C**	**2**	**4:0 conversion tract**
C2*	2	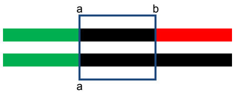
**D**	**12**	**Simple hybrid conversion tract (4:0/3:1 or 3:1/4:0/3:1)**
D1*	1	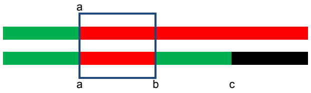
D2*	1	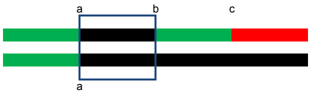
D3*	1	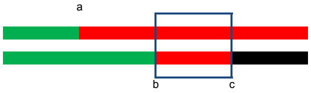
D4*	1	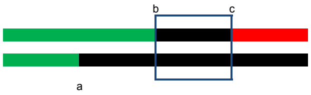
D5*	3	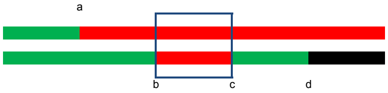
D6*	5	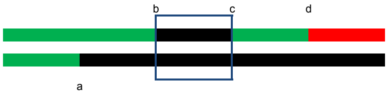
**E**	**5**	**Complex conversion tracts**
E2**	1	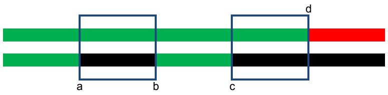
E3*	1	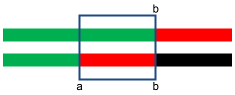
E5*	1	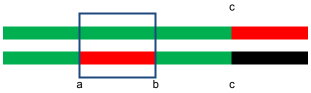
E11*	1	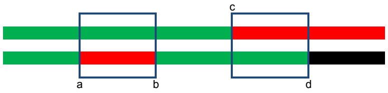
E26*	1	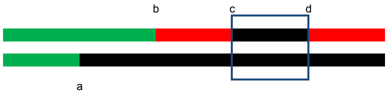

Novel 36

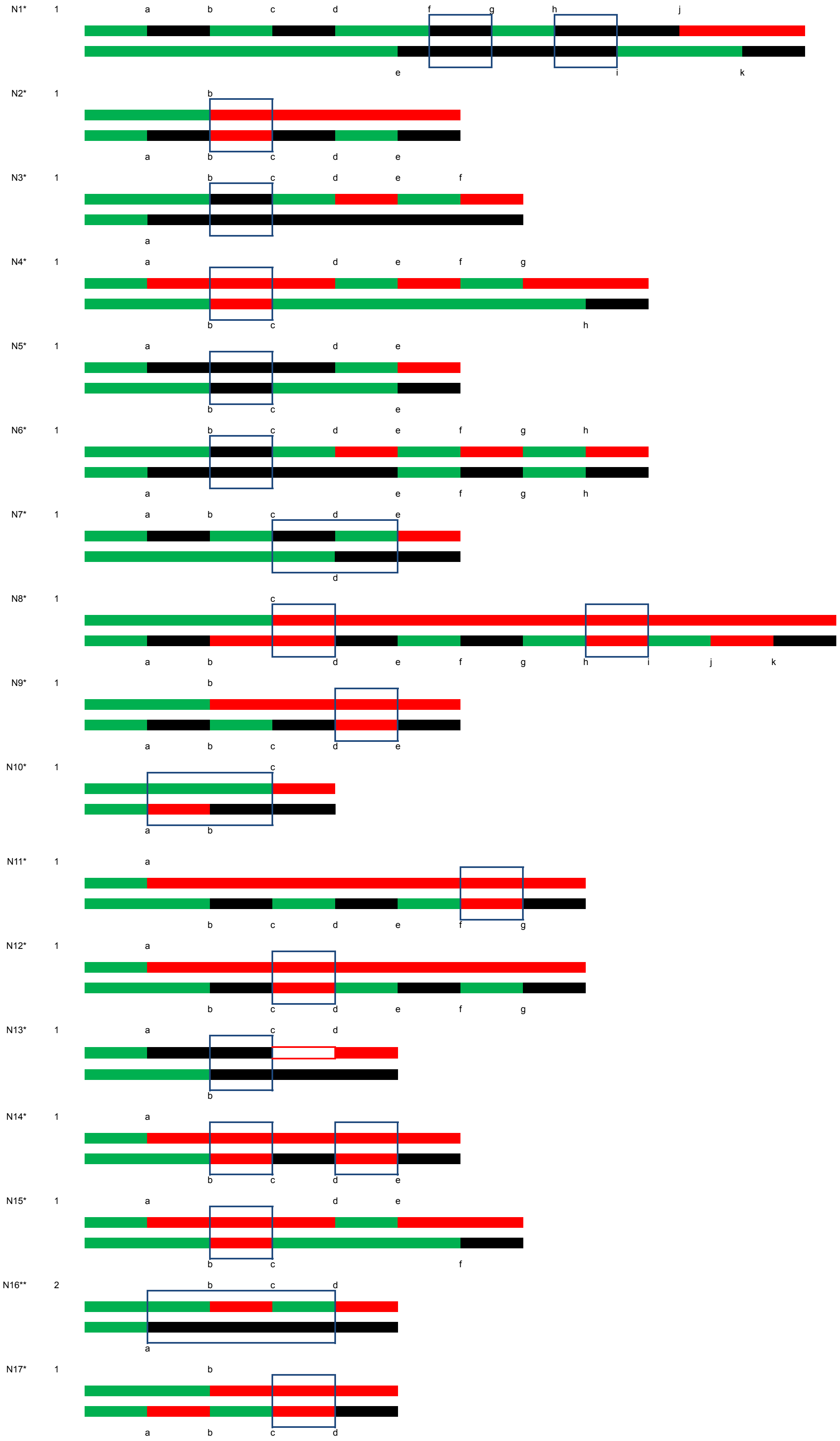
N1* 1
a b c d f g h j
e i k
N2* 1
b
a b c d e
N3* 1
b c d e f
a
N4* 1
a d e f g
b c h
N5* 1
a d e
b c e
N6* 1
b c d e f g h
a e f g h
N7* 1
a b c d e
d
N8* 1
c
a b d e f g h i j k
N9* 1
b
a b c d e
N10* 1
c
a b
N11* 1
a
b c d e f g
N12* 1
a
b c d e f g
N13* 1
a c d
b
N14* 1
a
b c d e
N15* 1
a d e
b c f
N16** 2
b c d
a
N17* 1
b
a b c d

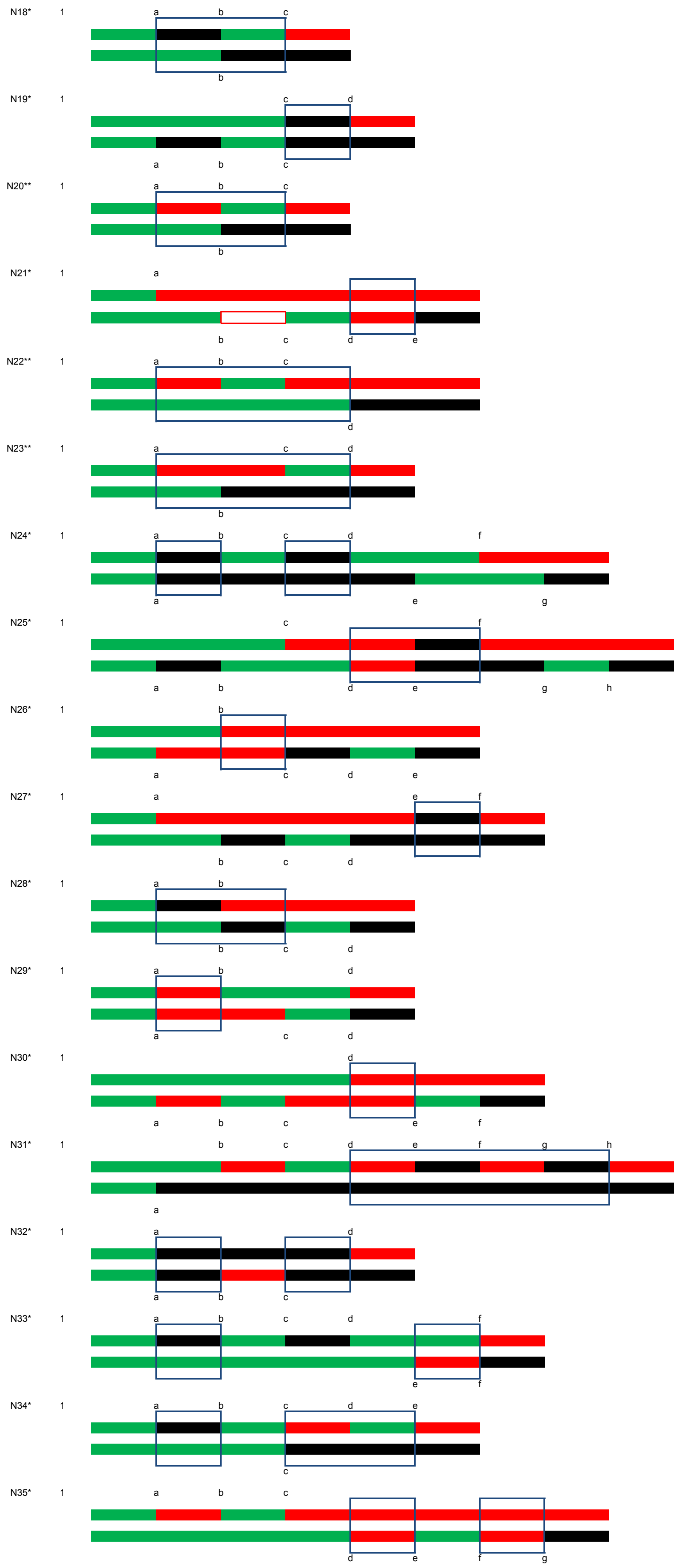

*G1-initiated DSB
** G2-initiated DSB
Green, red and black represent heterozygosity for SNPs, homozygosity for W303-1A SNPs, and homozygosity for YJM789 SNPs, respectively.
