# Supplementary material for: Genome-Destabilizing Effects Associated with Top1 Loss or Accumulation of Top1 Cleavage Complexes in Yeast
Source: PLoS Genet. 2015 Apr 1;11(4):e1005098. doi: 10.1371/journal.pgen.1005098 (PMC4382028; doi:10.1371/journal.pgen.1005098)
Supplement: S3 Table — See S1 Table legend for details. (DOCX) [file pgen.1005098.s004.docx]

**S3 Table. SGD coordinates for heterozygous and homozygous transitions on chromosome IV in CPT treated red/white sectors**

|  | Event Class^1^ | Transition Label^2^ | Markers flanking transitions^3^ | |
| --- | --- | --- | --- | --- |
| Sector |  |  | Left | Right |
| 1RW | D6 | a  b  c  d | 863830  866935  868904  890618 | 865277  867665  890618  892648 |
| 2RW | B2 | a  b | 841060  845945 | 841976  846463 |
| 3RW | A | a | 1017595 | 1036052 |
| 4RW | A | a | 1190182 | 1216161 |
| 5RW | B1 | a  b | 1261075  1264869 | 1262375  1267763 |
| 10RW | B1 | a  b | 1252616  1275176 | 1254293  1276330 |
| 11RW | B2 | a  b | 1368605  1370985 | 1370985  1374486 |
| 12RW | N36 | a  b  c  d  e  f  g | 830740  831863  832341  832830  839752  848693  852302 | 831863  832341  832583  833159  840535  849541  855308 |
| 13RW | N37 | a  b  c | 1157254  1188862  1189112 | 1161545  1189112  1189481 |
| 14RW | A | a | 1089446 | 1111969 |
| 15RW | N38 | a  b  c  d | 1366726  1367557  1372403  1374246 | 1367557  1368003  1374198  1374333 |
| 16RW | B2 | a  b | 1328086  1330538 | 1330102  1330866 |
| 17RW | A | a | 1089446 | 1111969 |
| 18RW | A | a | 509817 | 512801 |
| 19RW | B1 | a  b | 687816  701164 | 688339  701224 |
| 20RW | B2 | a  b | 1040924  1047033 | 1044556  1051325 |
| 21RW | B2 | a  b | 579229  587100 | 579742  587919 |
| 22RW | N20 | a  b  c | 1264435  1264937  1265332 | 1264855  1265186  1266551 |
| 23RW | B1 | a  b | 1039370  1040924 | 1040924  1044556 |
| 24RW | N39 | a  b  c  d  e  f | 1485798  1486159  1488015  1489226  1491125  1491920 | 1486091  1486789  1488287  1490813  1491302  1492290 |

^1^ Event Class: Classes of events are defined in Table S4.

^2^ Transition Label: These lower case letters reflect the transition from heterozygous to homozygous regions as shown in Table S4.

^3^ Markers flanking transition: SGD coordinates of SNPs located on each side of the transition.
