# Supplementary material for: Genome-Destabilizing Effects Associated with Top1 Loss or Accumulation of Top1 Cleavage Complexes in Yeast
Source: PLoS Genet. 2015 Apr 1;11(4):e1005098. doi: 10.1371/journal.pgen.1005098 (PMC4382028; doi:10.1371/journal.pgen.1005098)
Supplement: S4 Table — See S2 Table legend for details. (PDF) [file pgen.1005098.s005.pdf]

S4 Table. Conversion tracts associated with reciprocal crossovers in CPT-treated red/white sectors.

Class	Number observed		
**A**	**5**	**Simple crossover, no conversion tract**	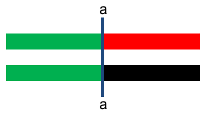
**B**	**9**	**3:1 conversion tract**	
B1**	4		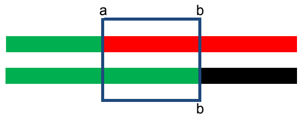
B2**	5		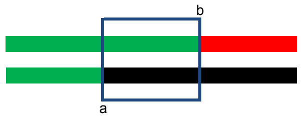
**D**	**1**	**Simple hybrid conversion tract (4:0/3:1 or 3:1/4:0/3:1)**	
D6*	1		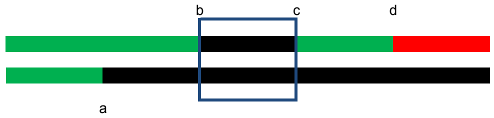
**Novel**	**5**		
N20**	1		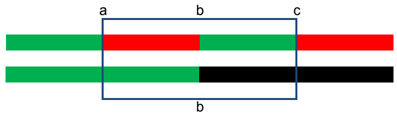
N36*	1		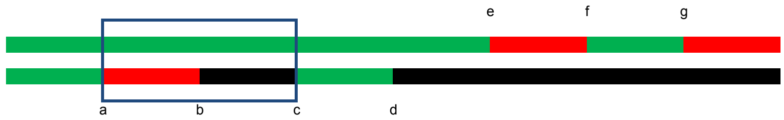
N37**	1		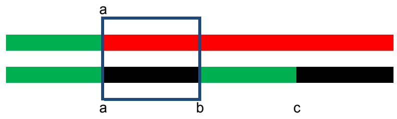
N38**	1		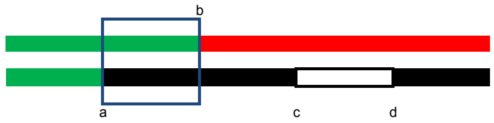
N39**	1		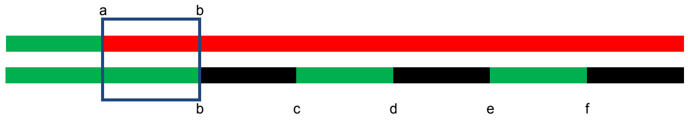

*G1-initiated DSB
** G2-initiated DSB
Green, red and black represent heterozygosity for SNPs, homozygosity for W303-1A SNPs, and homozygosity for YJM789 SNPs, respectively.
