# Supplementary material for: Genome-Destabilizing Effects Associated with Top1 Loss or Accumulation of Top1 Cleavage Complexes in Yeast
Source: PLoS Genet. 2015 Apr 1;11(4):e1005098. doi: 10.1371/journal.pgen.1005098 (PMC4382028; doi:10.1371/journal.pgen.1005098)
Supplement: S5 Table — See S1 Table legend for details. (DOCX) [file pgen.1005098.s006.docx]

**S5 Table. SGD coordinates for heterozygous and homozygous transitions on chromosome IV in *top1-T722A* red/white sectors**

| Sector | Event Class^1^ | Transition Label^2^ | Markers flanking transitions^3^ | | |
| --- | --- | --- | --- | --- | --- |
|  |  |  | Left | | Right |
| 1RW | B1 | a | 774716 | 775504 | |
|  |  | b | 785867 | 787240 | |
| 2RW | B2 | a | 839006 | 839752 | |
|  |  | b | 842052 | 842312 | |
| 4RW | B1 | a | 1171413 | 1173657 | |
|  |  | b | 1181603 | 1182080 | |
| 5RW | D5 | a | 546605 | 551730 | |
|  |  | b | 553854 | 554380 | |
|  |  | c | 555575 | 556844 | |
|  |  | d | 557100 | 558167 | |
| 7RW | A | a | 729738 | 730597 | |
| 8RW | N40 | a | 599224 | 601671 | |
|  |  | b | 608319 | 609891 | |
|  |  | c | 616610 | 617827 | |
|  |  | d | 620573 | 623017 | |
| 9RW | B2 | a | 1156998 | 1157254 | |
|  |  | b | 1173657 | 1171413 | |
| 10RW | A | a | 485657 | 495183 | |
| 11RW | B1 | a | 598460 | 598715 | |
|  |  | b | 608376 | 608572 | |
|  |  | c | 609138 | 611611 | |
| 12RW | A | a | 1394070 | 1397989 | |
| 13RW | B1 | a | 764803 | 764830 | |
|  |  | b | 1190182 | 1225482 | |
| 14RW | B1 | a | 1453193 | 1455114 | |
|  |  | b | 1459821 | 1460352 | |
| 15RW | B2 | a | 1171413 | 1173640 | |
|  |  | b | 1174594 | 1174705 | |
| 16RW | B2 | a | 1221462 | 1225482 | |
|  |  | b | 1333878 | 1336486 | |
| 17RW | A | a | 1235569 | 1240156 | |
| 18RW | B1 | a | 1283911 | 1284092 | |
|  |  | b | 1287823 | 1289866 | |
| 19RW | D6 | a | 701908 | 702466 | |
|  |  | b | 723149 | 724557 | |
|  |  | c | 725231 | 726748 | |
|  |  | d | 730720 | 731914 | |
| 21RW | B1 | a | 970231 | 971058 | |
|  |  | b | 978358 | 978621 | |
| 23RW | A | a | 1171413 | 1173657 | |
| 24RW | B1 | a | 1240303 | 1241971 | |
|  |  | b | 1249203 | 1251733 | |

^1^ Event Class: Classes of events are defined in Table S6.

^2^ Transition Label: These lower case letters reflect the transition from heterozygous to homozygous regions as shown in Table S6.

^3^ Markers flanking transition: SGD coordinates of SNPs located on each side of the transition.
