# Supplementary material for: Genome-Destabilizing Effects Associated with Top1 Loss or Accumulation of Top1 Cleavage Complexes in Yeast
Source: PLoS Genet. 2015 Apr 1;11(4):e1005098. doi: 10.1371/journal.pgen.1005098 (PMC4382028; doi:10.1371/journal.pgen.1005098)
Supplement: S7 Table — For each detected event, an event class (*) is listed that is correlated with S8 Table. ** Markers flanking transitions: SGD coordinates of SNPs located on each side of the transition. All reported coordinates are based on SGD coordinates from Feb. 2010 and may be different from those posted in SGD. (DOCX) [file pgen.1005098.s008.docx]

**S7 Table. SGD coordinates for events detected in sub-cultured clones.**

|  |  |  | **Clone**  **No.** |  | **Event**  **Class*** | **Markers flanking transitions**** | |
| --- | --- | --- | --- | --- | --- | --- | --- |
| **Strain** | **Genotype** | **Condition** |  | **Chr** |  | **Left** | **Right** |
| JSC25 | WT | YPD + DMSO | 29.1 | 13 | a1 | 681653 | 684380 |
|  |  |  |  |  |  | 684380 | 685125 |
|  |  |  |  |  |  | 685125 | 687473 |
|  |  |  | 30.1 | 10 | b1 | 149069 | 153061 |
|  |  |  |  |  |  | 157586 | 157675 |
|  |  |  |  |  |  | 157675 | 157805 |
|  |  |  |  |  |  | 157805 | 157905 |
|  |  |  |  |  |  | 157905 | 159185 |
|  |  |  |  |  |  | 161580 | 163352 |
|  |  | YPD + CPT | 20.1 | 2 | a2 | 102421 | 116691 |
|  |  |  |  | 3 | a3 | 260184 | 269360 |
|  |  |  |  | 8 | b2 | 216804 | 223781 |
|  |  |  |  |  |  | 225165 | 225901 |
|  |  |  |  | 11 | b2 | 176121 | 177475 |
|  |  |  |  |  |  | 205590 | 206366 |
|  |  |  |  | 12 | a1 | 962667 | 963376 |
|  |  |  | 21.1 | 12 | d1 | 447834 | 490725 |
|  |  |  | 22.1 | 7 | b2 | 138007 | 142576 |
|  |  |  |  |  |  | 146589 | 159699 |
|  |  |  |  | 9 | a2 | 277513 | 281875 |
| SLA46.D4 | *top1∆* | + *top1-T722A* plasmid | 1.1 | 8 | c1 | 211711 | 215271 |
|  |  |  |  |  |  | 215902 | 216553 |
|  |  |  | 2.1 | 8 | c1 | 211711 | 215271 |
|  |  |  |  |  |  | 215902 | 216553 |
|  |  |  |  | 12 | b3 | 447537 | 490725 |
|  |  |  |  |  |  | 493683 | 493832 |
|  |  |  |  | 12 | d1 | 606117 | 607697 |
|  |  |  |  |  |  | 608083 | 611242 |
|  |  |  |  | 12 | d1 | 665333 | 665741 |
|  |  |  |  |  |  | 665742 | 678184 |
|  |  |  | 3.1 | 4 | b3 | 1394070 | 1398890 |
|  |  |  |  |  |  | 1411220 | 1414657 |
|  |  |  |  | 7 | b3 | 122369 | 123323 |
|  |  |  |  |  |  | 130707 | 131363 |
|  |  |  |  | 8 | c2 | 211711 | 215271 |
|  |  |  |  |  |  | 216113 | 216553 |
|  |  |  |  | 12 | b3 | 545557 | 548317 |
|  |  |  |  |  |  | 552864 | 554927 |
|  |  |  |  | 12 | a1 | 1048627 | 1051101 |
|  |  |  | 4.1 | 8 | c1 | 211711 | 215271 |
|  |  |  |  |  |  | 216113 | 216553 |
|  |  |  |  | 11 | b2 | 83483 | 83729 |
|  |  |  |  |  |  | 84426 | 84604 |

* Event class: Classes of events are defined in Table S8.

** Markers flanking transitions: SGD coordinates of SNPs located on each side of the transition.
