# Supplementary material for: Genome-Destabilizing Effects Associated with Top1 Loss or Accumulation of Top1 Cleavage Complexes in Yeast
Source: PLoS Genet. 2015 Apr 1;11(4):e1005098. doi: 10.1371/journal.pgen.1005098 (PMC4382028; doi:10.1371/journal.pgen.1005098)
Supplement: S8 Table — The colored line represents a chromosome. Green, red and black represent heterozygosity for SNPs, homozygosity for W303-1A SNPs, and homozygosity for YJM789 SNPs, respectively. The lengths of chromosomes are not drawn to scale. (PDF) [file pgen.1005098.s009.pdf]

S8 Table. Chromosome depictions for events detected in clones sub-cultured 10 times.

Strain	Genotype	Condition	Event Class		Number observed	
**JSC25**	***WT***	**YPD + DMSO**	**a**		**1**	**Terminal LOH**
				a1	1	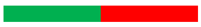
			**b**		**1**	**Gene Conversions**
				b1	1	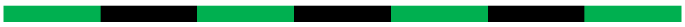
		**YPD + CPT**	**a**		**4**	**Terminal LOH**
				a1	1	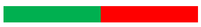
				a2	2	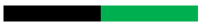
				a3	1	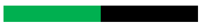
			**b**		**3**	**Gene Conversions**
				b2	3	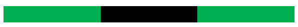
			**d**		**1**	**Terminal LOH at the rDNA locus (Chromosome XII)**
				d1	1	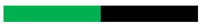
**SLA46.D4**	***top1*∆**	**+ *top1-T722A* plasmid**	**a**		**1**	**Terminal LOH**
				a1	1	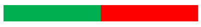
			**b**		**5**	**Gene Conversions**
				b2	1	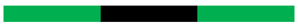
				b3	4	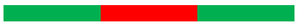
			**c**		**4**	**Amplifications and Deletions at *CUP1* locus (Chromosome VIII)**
				c1	3	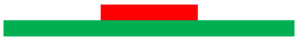
				c2	1	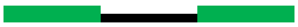
			**e**		**2**	**Amplifications and Deletions**
				e1	2	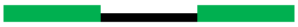

Green, red and black represent heterozygosity for SNPs, homozygosity for W303-1A SNPs, and homozygosity for YJM789 SNPs, respectively.
