# Supplementary material for: Genome-Destabilizing Effects Associated with Top1 Loss or Accumulation of Top1 Cleavage Complexes in Yeast
Source: PLoS Genet. 2015 Apr 1;11(4):e1005098. doi: 10.1371/journal.pgen.1005098 (PMC4382028; doi:10.1371/journal.pgen.1005098)
Supplement: S9 Table — Southern analysis data for the behavior of the CUP1 arrays in W303-1A and YJM789 are listed for all sub-cultured 1 and 10 clones. NC, # in blue and # in red represent no detectable change, estimated number of repeats lost, and estimated number of repeats gained, respectively. For CPT-treated and Top1-T722A sub-cultured clones, a column for instability is included, signifying that more than two bands were detected by Southern analysis. It should be noted that instability was only detected in these clones and not in untreated (WT) or top1Δ clones. (DOCX) [file pgen.1005098.s010.docx]

**S9 Table. *CUP1* Southern data for sub-cultured clones.**

|  |  |  |  | **Subculture 1** | | | **Subculture 10** | | | | | |
| --- | --- | --- | --- | --- | --- | --- | --- | --- | --- | --- | --- | --- |
| **Strain** | **Geno-type** | **Condition** | **Clone No.** | **Copy No. Status*** | | **Instability** | **Copy No. Status*** | | | | **Instability** | |
|  |  |  |  | **W303** | **YJM789** | **(Y/N)** | **W303** | | **YJM789** | | **(Y/N)** | |
| **JSC25** | **WT** | **YPD + DMSO** | 1 | **- 5** | NC | N | **-5** | | NC | | N | |
|  |  |  | 2 | NC | NC | N | NC | | NC | | N | |
|  |  |  | 3 | NC | NC | N | NC | | NC | | N | |
|  |  |  | 4 | NC | NC | N | NC | | NC | | N | |
|  |  |  | 5 | NC | NC | N | NC | | NC | | N | |
|  |  |  | 6 | NC | NC | N | NC | | NC | | N | |
|  |  |  | 8 |  |  |  | NC | | NC | | N | |
|  |  |  | 9 |  |  |  | NC | | NC | | N | |
|  |  |  | 11 |  |  |  | NC | | NC | | N | |
|  |  |  | 12 |  |  |  | NC | | **-1** | | N | |
|  |  |  | 13 | NC | NC | N |  | |  | |  | |
|  |  |  | 14 | NC | NC | N |  | |  | |  | |
|  |  |  | 15 | NC | NC | N |  | |  | |  | |
|  |  |  | 29.1 | NC | NC | N | **-2** | | NC | | N | |
|  |  |  | 30.1 |  |  |  | NC | | NC | | N | |
|  |  | **YPD** | 1 |  |  |  | NC | | NC | | N | |
|  |  |  | 2 |  |  |  | NC | | NC | | N | |
|  |  |  | 3 |  |  |  | NC | | NC | | N | |
|  |  |  | 4 |  |  |  | NC | | NC | | N | |
|  |  |  | 5 |  |  |  | NC | | NC | | N | |
|  |  |  | 6 |  |  |  | NC | | NC | | N | |
|  |  |  | 8 |  |  |  | **-3** | | NC | | N | |
|  |  |  | 9 |  |  |  | NC | | NC | | N | |
|  |  |  | 10 |  |  |  | NC | | NC | | N | |
|  |  |  | 11 |  |  |  | NC | | NC | | N | |
|  |  |  | 12 |  |  |  | NC | | NC | | N | |
|  |  |  | 33.1 |  |  |  | NC | | NC | | N | |
|  |  |  | 34.1 |  |  |  | NC | | NC | | N | |
|  |  | **YPD + CPT** | 1 | NC | **+1** | Y | **+5** | | NC | | N | |
|  |  |  | 2 | NC | NC | Y | **+5** | | NC | | N | |
|  |  |  | 3 | NC | NC | Y | **-2** | | **-2** | | N | |
|  |  |  | 4 | NC | NC | Y | **-2** | | **-2** | | N | |
|  |  |  | 5 | NC | NC | Y | **+5** | | **-4** | | N | |
|  |  |  | 6 | NC | NC | Y | **-3** | | **-4** | | N | |
|  |  |  | 7 |  |  |  | **-6** | | NC | | N | |
|  |  |  | 8 |  |  |  | **-6** | | NC | | N | |
|  |  |  | 12 |  |  |  | **-7** | | **-2** | | N | |
|  |  |  | 13 | NC | NC | Y |  | |  | |  | |
|  |  |  | 14 | NC | NC | Y |  | |  | |  | |
|  |  |  | 15 | NC | NC | Y |  | |  | |  | |
|  |  |  | 19.1 | NC | NC | Y | **-3** | | **-5** | | Y | |
|  |  |  | 20.1 |  |  |  | **-6** | | **-1** | | Y | |
|  |  |  | 21.1 |  |  |  | **-1** | | **-2** | | Y | |
|  |  |  | 22.1 |  |  |  | **-1** | | NC | | N | |
| **PG311** | **WT** | **YPD + DMSO** | 1 | NC | NC | N | NC | | NC | | N | |
|  |  |  | 2 | NC | NC | N | NC | | NC | | N | |
|  |  |  | 3 | NC | NC | N | NC | | NC | | N | |
|  |  |  | 4 | NC | NC | N | NC | | NC | | N | |
|  |  |  | 5 | NC | NC | N | NC | | NC | | N | |
|  |  |  | 6 |  |  |  | NC | | NC | | N | |
|  |  |  | 7 |  |  |  | NC | | **+4** | | N | |
|  |  |  | 8 |  |  |  | NC | | NC | | N | |
|  |  |  | 9 |  |  |  | NC | | NC | | N | |
|  |  |  | 10 |  |  |  | NC | | **+3** | | N | |
|  |  |  | 11 |  |  |  | NC | | NC | | N | |
|  |  |  | 12 |  |  |  | NC | | NC | | N | |
|  |  |  | 13 | **+2** | NC | N |  | |  | |  | |
|  |  |  | 14 | NC | NC | N |  | |  | |  | |
|  |  |  | 15 | NC | NC | N |  | |  | |  | |
|  |  |  | 19 |  |  |  | NC | | NC | | N | |
|  |  |  | 20 |  |  |  | NC | | NC | | N | |
|  |  |  | 21 |  |  |  | NC | | NC | | N | |
|  |  |  | 22 |  |  |  | **+2** | | NC | | N | |
|  |  |  | 23 |  |  |  | NC | | NC | | N | |
|  |  |  | 24 |  |  |  | NC | | NC | | N | |
|  |  |  | 27.1 |  |  |  | NC | | NC | | N | |
|  |  |  | 28.1 |  |  |  | **-5** | | NC | | N | |
|  |  | **YPD** | 1 |  |  |  | **+2** | | NC | | N | |
|  |  |  | 2 |  |  |  | **-6** | | NC | | N | |
|  |  |  | 3 |  |  |  | NC | | **-3** | | N | |
|  |  |  | 4 |  |  |  | NC | | NC | | N | |
|  |  |  | 5 |  |  |  | NC | | NC | | N | |
|  |  |  | 6 |  |  |  | NC | | NC | | N | |
|  |  |  | 7 |  |  |  | NC | | NC | | N | |
|  |  |  | 8 |  |  |  | NC | | NC | | N | |
|  |  |  | 9 |  |  |  | NC | | NC | | N | |
|  |  |  | 10 |  |  |  | NC | | NC | | N | |
|  |  |  | 11 |  |  |  | NC | | NC | | N | |
|  |  |  | 12 |  |  |  | NC | | NC | | N | |
|  |  |  | 19 |  |  |  | NC | | NC | | N | |
|  |  |  | 20 |  |  |  | NC | | NC | | N | |
|  |  |  | 21 |  |  |  | NC | | NC | | N | |
|  |  |  | 22 |  |  |  | **-1** | | NC | | N | |
|  |  |  | 23 |  |  |  | NC | | **+1** | | N | |
|  |  |  | 24 |  |  |  | NC | | NC | | N | |
|  |  | **YPD + CPT** | 1 | **-2** | NC | Y |  | |  | |  | |
|  |  |  | 2 | NC | NC | Y |  | |  | |  | |
|  |  |  | 3 | NC | NC | Y |  | |  | |  | |
|  |  |  | 4 | NC | NC | Y | **-2** | | NC | | N | |
|  |  |  | 5 | NC | NC | Y |  | |  | |  | |
|  |  |  | 6 |  |  |  | NC | | **-2** | | N | |
|  |  |  | 7 |  |  |  | **-3** | | **+1** | | N | |
|  |  |  | 8 |  |  |  | **-3** | | **-5** | | N | |
|  |  |  | 9 |  |  |  | **-4** | | **+4** | | N | |
|  |  |  | 10 |  |  |  | **-2** | | **+3** | | N | |
|  |  |  | 11 |  |  |  | **-2** | | NC | | N | |
|  |  |  | 13 | NC | NC | Y |  | |  | |  | |
|  |  |  | 14 | NC | NC | Y |  | |  | |  | |
|  |  |  | 15 | NC | NC | N |  | |  | |  | |
|  |  |  | 19 |  |  |  | NC | | NC | |  | |
|  |  |  | 20 |  |  |  | **-6** | | NC | | Y | |
|  |  |  | 21 |  |  |  | **-2** | | NC | | N | |
|  |  |  | 22 |  |  |  | **-2** | | **-3** | | N | |
|  |  |  | 23 |  |  |  | **-4** | | NC | | N | |
|  |  |  | 23.1 |  |  |  | **-2** | | NC | | Y | |
|  |  |  | 24 |  |  |  | **-6** | | NC | | N | |
|  |  |  | 24.1 | NC | NC | Y | **-1** | | **-1** | | Y | |
|  |  |  | 25.1 | NC | NC | Y | **+5** | | **+1** | | Y | |
| **SLA46.D4** | ***top1∆*** | **+ Vector plasmid** | 1 | NC | NC | N | NC | | NC | | N | |
|  |  |  | 2 | NC | NC | N | NC | | NC | | N | |
|  |  |  | 3 | NC | NC | N | NC | | NC | | N | |
|  |  |  | 4 | NC | NC | N | **-5** | | NC | | N | |
|  |  |  | 5 | NC | NC | N | **-5** | | NC | | N | |
|  |  |  | 6 | NC | NC | N | NC | | NC | | N | |
|  |  |  | 7 | NC | NC | N | NC | | NC | | N | |
|  |  |  | 8 | NC | NC | N | NC | | NC | | N | |
|  |  |  | 9 | NC | NC | N | **+2** | | NC | | N | |
|  |  |  | 10 | NC | NC | N | NC | | NC | | N | |
|  |  |  | 11 | NC | NC | N | NC | | NC | | N | |
|  |  |  | 12 | NC | NC | N | NC | | NC | | N | |
|  |  |  | 13 | NC | NC | N |  | |  | |  | |
|  |  |  | 13.1 |  |  |  | NC | | NC | | N | |
|  |  |  | 14 | NC | NC | N |  | |  | |  | |
|  |  |  | 14.1 |  |  |  | NC | | **+2** | | N | |
|  |  |  | 15 | NC | NC | N |  | |  | |  | |
|  |  |  | 16 | NC | NC | N |  | |  | |  | |
|  |  |  | 17.1 |  |  |  | NC | | NC | | N | |
|  |  |  | 39.1 |  |  |  | NC | | NC | | N | |
|  |  |  | 45.1 |  |  |  | NC | | NC | | N | |
|  |  |  | 46.1 |  |  |  | NC | | NC | | N | |
|  |  | **+ WT *TOP1* plasmid** | 1 | NC | NC |  |  | |  | |  | |
|  |  |  | 7 | NC | NC |  |  | |  | |  | |
|  |  |  | 7.1 |  |  |  | NC | | NC | | N | |
|  |  |  | 8 | NC | NC |  |  | |  | |  | |
|  |  |  | 8.1 |  |  |  | NC | | NC | | N | |
|  |  |  | 9 | NC | NC |  |  | |  | |  | |
|  |  |  | 9.1 |  |  |  | NC | | NC | | N | |
|  |  |  | 10 | NC | NC |  |  | |  | |  | |
|  |  |  | 10.1 |  |  |  | NC | | NC | | N | |
|  |  |  | 11.1 |  |  |  | NC | | NC | | N | |
|  |  |  | 12 | NC | NC |  |  | |  | |  | |
|  |  |  | 37.1 |  |  |  | NC | | NC | | N | |
|  |  |  | 38.1 |  |  |  | NC | | NC | | N | |
|  |  |  | 43.1 |  |  |  | NC | | NC | | N | |
|  |  |  | 44.1 |  |  |  | NC | | NC | | N | |
|  |  |  | 51.1 |  |  |  | NC | | NC | | N | |
|  |  |  | 52.1 |  |  |  | NC | | NC | | N | |
|  |  |  | 53.1 |  |  |  | NC | | NC | | N | |
|  |  |  | 54.1 |  |  |  | NC | | NC | | N | |
|  |  | **+ *top1-T722A* plasmid** | 1 | **+2** | NC | N |  | |  | |  | |
|  |  |  | 1.1 |  |  |  | NC | | NC | | N | |
|  |  |  | 2.1 |  |  |  | **+1** | | NC | | Y | |
|  |  |  | 3.1 |  |  |  | **-8** | | NC | | N | |
|  |  |  | 4.1 |  |  |  | NC | | NC | | Y | |
|  |  |  | 5 | **-5** | **-2** | Y |  | |  | |  | |
|  |  |  | 5.1 |  |  |  | **-1** | | **-1** | | Y | |
|  |  |  | 6 | **-2** | NC | N |  | |  | |  | |
|  |  |  | 6.1 |  |  |  | **-1** | | NC | | Y | |
|  |  |  | 7 | NC | NC | Y |  | |  | |  | |
|  |  |  | 8 | **-4** | NC | Y |  | |  | |  | |
|  |  |  | 9 | NC | NC | Y |  | |  | |  | |
|  |  |  | 10 | **+1** | NC | Y |  | |  | |  | |
|  |  |  | 11 | **+1** | NC | Y |  |  | |  | |  |
|  |  |  | 12 | NC | NC | Y |  | |  | |  | |
|  |  |  | 14 | NC | NC | Y |  | |  | |  | |
|  |  |  | 16 | NC | NC | N |  | |  | |  | |
|  |  |  | 35.1 |  |  |  | **-1** | | NC | | Y | |
|  |  |  | 36.1 |  |  |  | **-5** | | **-6** | | Y | |
|  |  |  | 41.1 |  |  |  | **-4** | | NC | | Y | |
|  |  |  | 42.1 |  |  |  | **-2** | | **+2** | | Y | |
|  |  |  | 47.1 |  |  |  | **+2** | | **-2** | | Y | |
|  |  |  | 48.1 |  |  |  | NC | | NC | | Y | |
|  |  |  | 49.1 |  |  |  | **+1** | | **-4** | | Y | |
|  |  |  | 50.1 |  |  |  | **+6** | | **-3** | | N | |

* Copy Number Status: The estimated number of *CUP1* repeats lost (blue) or gained (red).
