# Supplementary material for: Genome-Destabilizing Effects Associated with Top1 Loss or Accumulation of Top1 Cleavage Complexes in Yeast
Source: PLoS Genet. 2015 Apr 1;11(4):e1005098. doi: 10.1371/journal.pgen.1005098 (PMC4382028; doi:10.1371/journal.pgen.1005098)
Supplement: S10 Table — The total number of sub-cultured clones analyzed is in the rightmost column. The column labeled “Both” indicates the number of these clones that were heterozygous for the SNP located distal to the rDNA locus. We also show the number of sub-clones that were homozygous for the W303-1A- and YJM789-derived SNPs. (DOCX) [file pgen.1005098.s011.docx]

**S10 Table. Reciprocal crossovers at the rDNA locus after 10 sub-cultures.**

| **Strain** | **Geno-type** | **Conditions** | **SNP Distal to rDNA locus** | | | **Total Clones Analyzed** |
| --- | --- | --- | --- | --- | --- | --- |
|  |  |  | **Both** | **W303-1A only** | **YJM789 only** |  |
| JSC25 | WT | YPD | 40 | 0 | 0 | 40 |
|  |  | YPD + DMSO | 21 | 0 | 0 | 21 |
|  |  | YPD + CPT | 23 | 15 | 5 | 43 |
|  |  |  |  |  |  |  |
| SLA46.D4 | *top1∆* | + Vector plasmid | 24 | 14 | 6 | 44 |
|  |  | + WT *TOP1* plasmid | 30 | 7 | 7 | 44 |
|  |  | + *top1-T722A* plasmid | 10 | 22 | 12 | 44 |
